# Frailty and risk of microvascular complications in patients with type 2 diabetes: a population-based cohort study

**DOI:** 10.1186/s12916-022-02675-9

**Published:** 2022-12-08

**Authors:** Yuanjue Wu, Ting Xiong, Xiao Tan, Liangkai Chen

**Affiliations:** 1 Department of Nutrition and Food Hygiene, Hubei Key Laboratory of Food Nutrition and Safety, School of Public Health, Tongji Medical College, Huazhong University of Science and Technology, 13 Hangkong Rd, Wuhan, 430030 China; 2 Department of Clinical Nutrition, Union Hospital, Tongji Medical College, Huazhong University of Science and Technology, Wuhan, China; 3 Department of Nutrition and Food Hygiene, School of Public Health, Guangzhou Medical University, Guangzhou, China; 4 Department of Medical Sciences, Uppsala University Hospital, Entrance 40, 75185 Uppsala, Sweden; 5 Department of Clinical Neuroscience, Karolinska Institute, Stockholm, Sweden; 6 Ministry of Education Key Lab of Environment and Health, School of Public Health, Tongji Medical College, Huazhong University of Science and Technology, Wuhan, China

**Keywords:** Frailty, Type 2 diabetes, Diabetic microvascular complications, Cohort, UK Biobank

## Abstract

**Background:**

Cross-sectional studies found that frailty was associated with prevalent diabetic microvascular complications (DMC). Longitudinal evidence in this regard is inconclusive and insufficient. We aimed to prospectively evaluate the association of pre-frailty and frailty with DMC in patients with type 2 diabetes (T2D).

**Methods:**

We included 18,062 adults (mean age 59.4 ± 7.2 years, 37.4% female) with T2D at baseline in the UK Biobank. Frailty was defined using the frailty phenotype according to five components (weight loss, exhaustion, low physical activity, slow gait speed, and low grip strength). DMC, defined as diabetic nephropathy, diabetic neuropathy, or diabetic retinopathy, was identified using hospital inpatient records and death registries. Cox proportional hazard regression models considering competing risks were used to evaluate the associations of frailty phenotype with overall DMC events and subtypes.

**Results:**

Among all participants, 6101 (33.8%) were classified as non-frail, 10,073 (55.8%) were classified as pre-frail, and 1888 (10.4%) were classified as frail. During a median follow-up of 12.0 years, 3678 DMC cases were documented, including 2213 diabetic nephropathy, 1520 diabetic retinopathy, and 673 diabetic neuropathy events. In the multivariable-adjusted model, compared with participants with non-frail, both pre-frailty and frailty were significantly associated with increased risk of overall DMC (HR 1.10, 95% CI: [1.02, 1.18] for pre-frailty and HR 1.52 [95% CI: 1.36, 1.69] for frailty). Similar results were observed in the subtypes of DMC. For each one-point increase in frailty phenotype score, the risk of overall DMC, diabetic nephropathy, diabetic retinopathy, and diabetic neuropathy event increased by 13%, 16%, 10%, and 20%, respectively.

**Conclusions:**

Both pre-frailty and frailty were associated with an increased risk of DMC in patients with T2D. These findings have important implications for integrating early assessment and surveillance of frailty in diabetes and may favor the identification of at-risk patients.

**Supplementary Information:**

The online version contains supplementary material available at 10.1186/s12916-022-02675-9.

## Background

Diabetes is one of the fastest-growing global health issues of the twenty-first century, with 537 million adults worldwide now living with diabetes in 2021, and this number is projected to reach 783 million in 2045 [1]. Diabetic microvascular complications (DMC) have emerged as major aftermath of the worldwide diabetes pandemic, leading to loss of visual, renal, and neurologic functions, with profound effects on quality of life, demand for health services, and economic costs [2, 3]. DMC prevalence is high in patients with diabetes, affecting half of the patients with type 2 diabetes (T2D) [4]. About 25% of patients with diabetes will develop diabetic nephropathy [5], almost 34.6% will develop diabetic retinopathy [6], and nearly 50% will develop diabetic neuropathy [7]. Given the high incidence and serious consequences, early recognition and a better understanding of the risk factors for DMC are needed.

Frailty, an emerging public health concern worldwide paralleled with population aging, is characterized by a decline in functioning across multiple physiological systems, with a resultant increased susceptibility to stressors [8, 9]. This condition increases the risk of a range of adverse health outcomes, including disability, falls, fracture, lower quality of life, loneliness, cognitive decline, hospital admission, and mortality [9]. Recently, frailty has attracted widespread attention in the field of diabetes. The prevalence of frailty and pre-frailty in community-dwelling older adults with diabetes was 20.1% and 49.1%, respectively, and adults with diabetes were more susceptible to frailty than those without diabetes [10]. Frailty further increases the risk of adverse outcomes, including disability [11, 12], fractures [13], hospitalization [12, 14, 15], major cardiovascular events [14, 16], and mortality [12, 14, 15] in diabetes.

A cross-sectional study of 146 inpatients aged 60 years or older with T2D found that both pre-frailty and frailty were significantly associated with diabetic nephropathy (odds ratio 4.31 and 4.46, respectively) [17]. Another cross-sectional study of 292,170 individuals with diabetes (mean age 64.7 years) found that frailty was associated with prevalent DMC [18]. However, longitudinal evidence in this regard is inconclusive and insufficient. Two prospective studies suggested that the presence of frailty (defined by the Rockwood frailty index and Clinical Frailty Scale) was independently associated with the risk of overall DMC [16, 19]. However, a population-based, retrospective study found that frailty derived from Johns Hopkins-adjusted clinical groups (weighted comorbidity score identified from electronic medical records) was not associated with overall DMC [20]. Frailty phenotype, described by Fried and colleagues who consider frailty as a clinical presentation of weight loss, weakness, exhaustion, slowness, and low physical activity level [21], has been a dominant criterion in the frailty literature. Compared with other criteria, the frailty phenotype provided a potential standardized definition for frailty, and the clear criteria are relatively easy and inexpensive to apply [21], which have been validated as consistent with a medical syndrome linked to distinct biology [9]. However, to our knowledge, no studies have investigated the association of frailty phenotype with the incidence of overall DMC and its subtypes (diabetic nephropathy, diabetic retinopathy, and diabetic neuropathy). Therefore, to fill these knowledge gaps, we explore the relationship between frailty phenotype and incident DMC in patients with T2D.

## Methods

### Study population

The UK Biobank is a large population-based cohort of more than 500,000 participants aged 40−69 years, recruited in one of 22 assessment centers between 2006 and 2010 in England, Scotland, and Wales [22]. Participants were invited to complete touchscreen questionnaires, have physical measurements, and provide biological samples at the baseline. All participants provided informed consent, and the study was approved by the North West–Haydock Research Ethics Committee (16/NW/0274).

Prevalent T2D at baseline was identified using UK Biobank algorithms by Eastwood et al. through hospital inpatient records, self-reported medical history, and medication, which is a reliable measurement with 96% accuracy [23]. We further considered the biochemical examination for glycated hemoglobin A1c (HbA1c) based on the algorithms. We used the cutoffs of HbA1c for defining diabetes, 48 mmol/mol (6.5%). The definitions for prevalent diabetes are presented in Additional file 1: Table S1. We compared the date of the patients’ first-time diabetes diagnosis and the date of entry to the study. Those who had self-reported or were diagnosed with diabetes before or on the cohort entry were recognized as baseline diabetes. We excluded participants who withdrew their information (*n* = 99). Those without diabetes at baseline (*n* = 471,048), who had been diagnosed with type 1 diabetes or diagnosed with diabetes before 30 years old (*n* = 3434), had DMC at baseline (*n* = 2983), and those who had no data for frailty phenotype were further excluded (*n* = 6879). Finally, 18,062 individuals with T2D were included in the present analysis (Additional file 1: Fig. S1).

### Assessment of frailty phenotype

The original definition of frailty phenotype was described and applied in the Cardiovascular Health Study by Fried and colleagues [21], and the items have been adapted in the UK Biobank [24, 25]. Weight loss was self-reported as the question “Compared with one year ago, has your weight changed?” (response: yes, lost weight = 1; others = 0). Exhaustion was self-reported according to the question “Over the past 2 weeks, how often have you felt tired or had little energy?” (response: more than half the days or nearly every day = 1; others = 0). Physical activity was assessed using the International Physical Activity Questionnaire short form, which computed the sum of walking, moderate activity, and vigorous activity to estimate the total metabolic equivalents (MET) minutes per week. Physical activity was categorized into quintiles of sex- and age-specific levels of total MET minutes per week, in which the lowest quintile was identified as “low physical activity.” Slow gait speed was self-reported with the question “How would you describe your usual walking pace?” (response: slow = 1; others = 0). Hand grip strength was measured by using a Jamar J00105 hydraulic hand dynamometer. The measured grip strength was expressed in kilograms by sex- and BMI-adjusted, and the cutoff points have referenced the points by Fried and colleagues [21]. The detailed definition of frailty phenotype was described in Additional file 1: Table S2. Participants were classified as frail (fulfilled ≥ 3 criteria), pre-frail (fulfilled 1–2 criteria), or non-frail (fulfilled 0 criteria).

### Ascertainment of outcomes

The primary outcome of interest was overall incident DMC, a composite indicator of the first occurrence of diabetic nephropathy, diabetic neuropathy, and/or diabetic retinopathy. Secondary outcomes included the incidence of three DMC subtypes (diabetic nephropathy, diabetic neuropathy, and diabetic retinopathy). DMC was identified using cumulative hospital inpatient records and death record linkage to national death registries. The definition was described according to the 9th and 10th revisions of the International Classification of Diseases (ICD-9 and ICD-10) and self-reported data fields with choice-, disease-, or procedure-specific codes (Additional file 1: Table S3). We compared the date of the first diagnosis of DMC with the baseline date to distinguish between baseline and incident DMC. Participants diagnosed with DMC after the baseline visit date were identified as incident DMC. At the time of analysis, hospital admission data was available until 30 September 2021 for England, 31 July 2021 for Scotland, and 28 February 2018 for Wales.

### Covariates

Sociodemographic factors, lifestyle factors, and health and medical history were acquired using touch screen questionnaires at the baseline. Socioeconomic deprivation was evaluated by Townsend deprivation index scores, and higher scores represent higher levels of socioeconomic deprivation [26]. We defined a healthy diet score based on dietary priorities for cardiovascular disease, diabetes, and obesity [27]. A higher score indicates healthier dietary habits. Definitions of each component of a healthy diet score were described in Additional file 1: Table S4. Smoking status was categorized as ever, former, or current smokers. Alcohol consumption was calculated based on the frequency and alcohol equivalent of different drinks consumed on a typical day/week/month. Height, weight, and blood pressure were measured by a trained nurse during the initial assessment center visit. Body mass index (BMI) was determined as weight in kilograms divided by the square of height in meters. Mean arterial pressure (MAP) was calculated by the following formula: MAP = diastolic blood pressure + 1/3(systolic blood pressure − diastolic blood pressure). HbA1c, lipid profiles (total cholesterol [TC], low-density lipoprotein cholesterol [LDL-C], high-density lipoprotein cholesterol [HDL-C], and triglycerides [TG]), serum creatinine, and cystatin C were measured in the blood sample collected at recruitment. The estimated glomerular filtration rate (eGFR) was calculated from serum creatinine and cystatin C [28]. Considering that long-term chronic diseases are associated with frailty [24], we included a number of long-term conditions [24, 29] as covariates. Given that this study was conducted in patients with diabetes, we exclude diabetes from the number of long-term conditions. The detailed definitions of long-term conditions are shown in Additional file 1: Table S5.

### Statistical analysis

Cox proportional hazard regression models considering competing risks (non-DMC-related deaths) by using the cause-specific hazard function model [30] were used to evaluate the associations of frailty phenotype with overall DMC events and subtypes (diabetic nephropathy, diabetic neuropathy, or diabetic retinopathy). The results were presented as hazard ratios (HRs) with 95% confidence intervals (CIs). The time to events was calculated from the date of baseline recruitment to the date of first-time DMC diagnosis, lost to follow-up, death, or the censoring date (30 September 2021 for England, 31 July 2021 for Scotland, 28 February 2018 for Wales), whichever occurred first. Frailty phenotype was assessed as a categorical variable (frail, pre-frail, or non-frail), and the category of “non-frail” was set as the referent in each model or a continuous variable. Frailty phenotype scores were used as a continuous variable in multivariate models when testing the linear trend (per frailty phenotype score increase). The dose–response shape of the association of frailty phenotype score with incident DMC events and subtypes was presented by using the restricted cubic spline model (rms, hmisc, lattice, and survival packages in the R software).

We considered the following covariates in multivariable models sequentially. Model 1 adjusted for age (continuous), sex (male or female), ethnicity (White, mixed, Asian, Black, Chinese, others, or unknown), educational attainment (college or university, vocational, upper secondary, lower secondary, others, or unknown), Townsend deprivation index (in quintiles), annual household income (< 18,000, 18,000–30,999, 31,000–51,999, 52,000–100,000, or >100,000£), and assessment centers (22 categories). Model 2 additionally adjusted for smoking status (ever, former, or current smokers), alcohol intake (0, 0.1–4.9, 5.0–14.9, 15.0–19.9, 20.0–29.9, or ≥ 30.0 g/day), healthy diet score (in quintiles), and BMI (< 18.5, 18.5–24.9, 25.0–29.9, 30.0–34.9, or ≥ 35.0 kg/m^2^). Model 3 further adjusted for no. of long-term conditions (0, 1, 2, 3, 4, and ≥ 5). Diabetes duration (< 1, 1–4, 5–9, ≥ 10 years), HbA1c (< 53, ≥ 53 mmol/mol [7%]), diabetes medication use (none, only oral medication, only insulin, or insulin and oral medication), lipid-lowering treatment, antihypertensive medication use, and aspirin use were additionally included in the fully adjusted model (model 4). Missing values were considered as dummy variables in regression models. The analyses for five frailty components and DMC events were further adjusted for other frailty components (mutual adjustment).

Several secondary analyses were performed. First, we conducted the stratified analyses that examined the associations of frailty phenotype and overall DMC events across age, sex, educational attainment, Townsend deprivation index, annual household income, smoking status, healthy diet score, BMI, diabetes duration, diabetes medication use, HbA1c levels, and no. of long-term conditions. The joint test was used to examine the interactions between frailty and these subgroups [31]. Second, we excluded those diagnosed with DMC or who died within 2 years of follow-up to minimize the reverse causality. Third, sensitivity analyses were conducted by sequentially adjusting for lipid profile, MAP, and eGFR at baseline based on model 4. Fourth, we used the Fine and Gray subdistribution hazard model, an alternative approach to taking competing risk of mortality, to examine the associations between frailty and DMC. Fifth, sensitivity analyses were conducted by using an alternative measure of comorbidities, the Charlson Comorbidity Index [32] (Additional file 1: Table S6). We also performed mediation analyses to explore the effect of no. of long-term conditions or Charlson Comorbidity Index on the association between frailty and the risk of DMC. It was conducted by using the SAS mediate macro written by Hertzmark et al. [33].

Statistical analyses were performed using SAS version 9.4 (SAS Institute Inc., Cary, NC, USA), and the restricted cubic spline model was conducted in the R software (the R Foundation, http://www.r-project.org, version 4.1.2). *P*-value < 0.05 was considered statistically significant (two-sided tests).

## Results

### Baseline characteristics

We included 18,062 middle-aged and older adults with diabetes (mean age 59.4 ± 7.2 years, 37.4% female), of whom 6101 (33.8%) were classified as non-frail, 10,073(55.8%) were classified as pre-frail, and 1888 (10.4%) were classified as frail (Table 1). Compared with subjects with non-frailty, those with pre-frailty and frailty were more likely to have higher BMI, current smokers, lower educated, lower income, less physical activity, and less alcohol intake. They were also more prone to have a longer duration of diabetes, higher HbA1c levels, more prevalent long-term conditions, use of diabetes medication, antihypertensive medication, lipid-lowering treatment, and aspirin at baseline.

**Table 1:** Baseline characteristics of 18,062 patients with type 2 diabetes by frailty phenotype

Variables	Overall	Frailty phenotype			*P* value
		Non-frail	Pre-frail	Frail	
Number of participants	18,062	6101 (33.8%)	10,073 (55.8%)	1888 (10.4%)	
Age, mean (SD), years	59.4 (7.2)	59.6 (7.1)	59.3 (7.3)	59.5 (7.1)	0.074
BMI, mean (SD), kg/m^2^	31.3 (5.7)	30.0 (5.0)	31.6 (5.7)	34.0 (6.7)	< 0.001
Male, *n* (%)	11,311 (62.6%)	4119 (67.5%)	6226 (61.8%)	966 (51.2%)	< 0.001
White ethnicity, *n* (%)	16,027 (88.7%)	5564 (91.2%)	8840 (87.8%)	1623 (86.0%)	< 0.001
Townsend deprivation index, median (IQR)	− 1.5 (− 3.3 to 1.6)	− 2.1 (− 3.6 to 0.7)	− 1.4 (− 3.2 to 1.7)	0.0 (− 2.5 to 3.2)	< 0.001
Education, *n* (%)					< 0.001
College or university	4855 (26.9%)	1807 (29.6%)	2688 (26.7%)	360 (19.1%)	
Vocational	2664 (14.7%)	978 (16.0%)	1429 (14.2%)	257 (13.6%)	
Upper secondary	1796 (9.9%)	622 (10.2%)	1010 (10.0%)	164 (8.7%)	
Lower secondary	4461 (24.7%)	1442 (23.6%)	2590 (25.7%)	429 (22.7%)	
Others	4071 (22.5%)	1198 (19.6%)	2233 (22.2%)	640 (33.9%)	
Unknown	215 (1.2%)	54 (0.9%)	123 (1.2%)	38 (2.0%)	
Annual household income, *n* (%), £					< 0.001
< 18,000	5073 (28.1%)	1358 (22.3%)	2896 (28.8%)	819 (43.4%)	
18,000–30,999	4410 (24.4%)	1524 (25.0%)	2478 (24.6%)	408 (21.6%)	
31,000–51,999	3424 (19.0%)	1339 (21.9%)	1868 (18.5%)	217 (11.5%)	
52,000–100,000	2257 (12.5%)	922 (15.1%)	1234 (12.3%)	101 (5.3%)	
> 100,000	477 (2.6%)	194 (3.2%)	262 (2.6%)	21 (1.1%)	
Unknown	2421 (13.4%)	764 (12.5%)	1335 (13.3%)	322 (17.1%)	
Smoking status, *n* (%)					< 0.001
Never	7949 (44.0%)	2678 (43.9%)	4480 (44.5%)	791 (41.9%)	
Former	8029 (44.5%)	2816 (46.2%)	4449 (44.2%)	764 (40.5%)	
Current	2008 (11.1%)	584 (9.6%)	1104 (11.0%)	320 (16.9%)	
Unknown	76 (0.4%)	23 (0.4%)	40 (0.4%)	13 (0.7%)	
Alcohol intake, median (IQR), g/day	9.7 (1.6 to 21.3)	11.9 (3.5 to 25.5)	8.7 (1.2 to 20.5)	2.8 (0.0 to 13.9)	< 0.001
Healthy diet score, mean (SD)	3.0 (2.0 to 4.0)	3.0 (2.0 to 4.0)	3.0 (2.0 to 4.0)	3.0 (2.0 to 4.0)	< 0.001
Physical activity, median (IQR), MET-h/week	23.1 (9.6 to 50.9)	33.5 (17.5 to 64.5)	21.3 (8.2 to 47.5)	5.0 (1.1 to 13.3)	< 0.001
Diabetes duration, *n* (%), years					< 0.001
< 1	4757 (26.3%)	1698 (27.8%)	2648 (26.3%)	411 (21.8%)	
1–4.9	6152 (34.1%)	1983 (32.5%)	3508 (34.8%)	661 (35.0%)	
5–9.9	4408 (24.4%)	1517 (24.9%)	2421 (24.0%)	470 (24.9%)	
≥ 10	2745 (15.2%)	903 (14.8%)	1496 (14.9%)	346 (18.3%)	
Diabetes medication use, *n* (%)					< 0.001
None	8192 (45.4%)	3037 (49.8%)	4467 (44.3%)	688 (36.4%)	
Only oral medication	8093 (44.8%)	2458 (40.3%)	4664 (46.3%)	971 (51.4%)	
Only insulin	671 (3.7%)	293 (4.8%)	318 (3.2%)	60 (3.2%)	
Insulin and oral medication	1106 (6.1%)	313 (5.1%)	624 (6.2%)	169 (9.0%)	
HbA1c, *n* (%), mmol/mol					0.0360
< 53.0	11,019 (61.0%)	3748 (61.4%)	6163 (61.2%)	1108 (58.7%)	
≥ 53.0	5980 (33.1%)	2008 (32.9%)	3331 (33.1%)	641 (34.0%)	
Missing	1063 (5.9%)	345 (5.7%)	579 (5.7%)	139 (7.4%)	
Antihypertensive medication use, *n* (%)	10,361 (57.4%)	3222 (52.8%)	5860 (58.2%)	1279 (67.7%)	< 0.001
Lipid-lowering treatment, *n* (%)	12,420 (68.8%)	3966 (65.0%)	7005 (69.5%)	1449 (76.7%)	< 0.001
Aspirin use, *n* (%)	7868 (43.6%)	2558 (41.9%)	4439 (44.1%)	871 (46.1%)	0.002
No. of long-term conditions, *n* (%)					< 0.001
0	2853 (15.8%)	1234 (20.2%)	1510 (15.0%)	109 (5.8%)	
1	6084 (33.7%)	2357 (38.6%)	3370 (33.5%)	357 (18.9%)	
2	4874 (27.0%)	1559 (25.6%)	2793 (27.7%)	522 (27.6%)	
3	2553 (14.1%)	683 (11.2%)	1463 (14.5%)	407 (21.6%)	
4	1084 (6.0%)	188 (3.1%)	628 (6.2%)	268 (14.2%)	
≥ 5	614 (3.4%)	80 (1.3%)	309 (3.1%)	225 (11.9%)	
DMC cases, *n* (%)	3678 (20.4%)	1067 (17.5%)	2031 (20.2%)	580 (30.7%)	< 0.001
One complication	3036 (16.8%)	911 (14.9%)	1666 (16.5%)	459 (24.3%)	< 0.001
Nephropathy	2213 (12.3%)	588 (9.6%)	1235 (12.3%)	390 (20.7%)	< 0.001
Retinopathy	1520 (8.4%)	481 (7.9%)	847 (8.4%)	192 (10.2%)	0.008
Neuropathy	673 (3.7%)	168 (2.8%)	366 (3.6%)	139 (7.4%)	< 0.001
Two complications	556 (3.1%)	142 (2.3%)	313 (3.1%)	101 (5.3%)	< 0.001
Nephropathy + retinopathy	383 (2.1%)	92 (1.5%)	225 (2.2%)	66 (3.5%)	< 0.001
Nephropathy + neuropathy	248 (1.4%)	51 (0.8%)	141 (1.4%)	56 (3.0%)	< 0.001
Neuropathy + retinopathy	183 (1.0%)	41 (0.7%)	103 (1.0%)	39 (2.1%)	< 0.001
Three complications	86 (0.5%)	14 (0.2%)	52 (0.5%)	20 (1.1%)	< 0.001

*Abbreviations*: *BMI* body mass index, *DMC* diabetic microvascular complication, *HbA1c* glycated hemoglobin A1c, *MET* metabolic equivalent, *IQR* interquartile range, *SD* standard deviation

During a median follow-up of 12.0 years (198,711 person-years), 3678 DMC cases were documented, including 2213 diabetic nephropathy, 1520 diabetic retinopathy, and 673 diabetic neuropathy events. Of the 3678 new complications, 3036, 556, and 86 patients had one, two, and three DMC, respectively (Table 1). Compared with subjects with one DMC, those with two and three DMC were more likely to be younger and male, have higher BMI, and be subjected to greater deprivation. They were also more prone to have a longer duration of diabetes, higher HbA1c levels, and more common use of diabetes medication, antihypertensive medication, lipid-lowering treatment, and aspirin at baseline (Additional file 1: Table S7).

### Association between frailty phenotype and risk of DMC

**Fig. 1:**
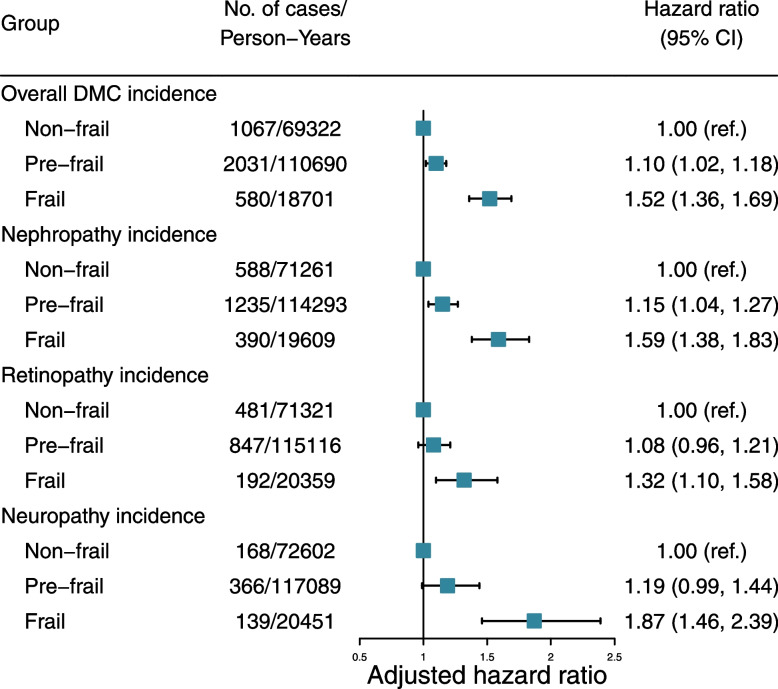
Association between frailty phenotype and risk of diabetic microvascular complications. Cox proportional hazards models adjusted for age, sex, ethnicity, educational attainment, Townsend deprivation index, annual household income, assessment centers, smoking status, alcohol intake, healthy diet score, BMI, no. of long-term conditions, diabetes duration, HbA1c, diabetes medication use, lipid-lowering treatment, antihypertensive medication use, and aspirin use

**Fig. 2:**
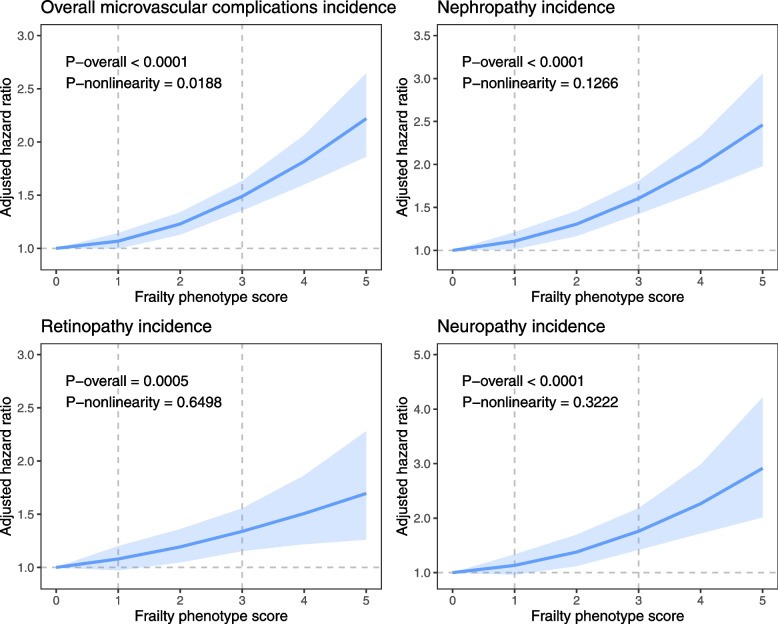
Dose-response curves for frailty phenotype scores and incidence of diabetic microvascular complications. Data are presented as adjusted hazard ratios with the 95% confidence interval shown as shading. The restricted cubic spline models adjusted for age, sex, ethnicity, educational attainment, Townsend deprivation index, annual household income, assessment centers, smoking status, alcohol intake, healthy diet score, BMI, no. of long-term conditions, diabetes duration, HbA1c, diabetes medication use, lipid-lowering treatment, antihypertensive medication use, and aspirin use

The incidence of overall DMC per 1000 person-years for non-frail, pre-frail, and frail was 15.39, 18.35, and 31.01, respectively. In Cox regression analyses, we observed that both pre-frailty and frailty were independently and significantly associated with increased risk of DMC and subtypes (Fig. 1 and Additional file 1: Table S8). In the fully adjusted model (model 4), compared with participants with non-frailty, the HRs of those with pre-frailty and frailty were 1.10 (95% CI: 1.02, 1.18) and 1.52 (95% CI: 1.36, 1.69), respectively (*P* for trend < 0.0001). Similarly, compared to those without frailty, patients with pre-frailty and frailty had a higher risk of developing diabetic nephropathy events (HR 1.15 [95% CI: 1.04, 2.27] for pre-frailty and HR 1.59 [95% CI: 1.38, 1.83] for frailty). Compared to those without frailty, patients with frailty had a higher risk of developing diabetic retinopathy events (HR 1.32 [95% CI: 1.10, 1.58]) and diabetic neuropathy events (HR 1.87 [95% CI: 1.46, 2.39]). In restricted cubic splines, we observed a positive non-linear relationship between frailty phenotype score and incidence of overall DMC (*P*_overall_ < 0.0001 and *P*_non-linear_ = 0.0188) and a linear relationship between frailty phenotype score and incidence of DMC subtypes (all *P*_overall_ < 0.001 and *P*_non-linear_ > 0.05, Fig. 2). For each one-point increase in frailty phenotype score, the risk of overall DMC, diabetic nephropathy, diabetic retinopathy, and diabetic neuropathy event increased by 13% (95% CI: 10%, 17%), 16% (95% CI: 11%, 20%), 10% (95% CI: 5%, 16%), and 20% (95% CI: 12%, 29%), respectively (Additional file 1: Table S8).

### Association between frailty components and risk of DMC

**Fig. 3:**
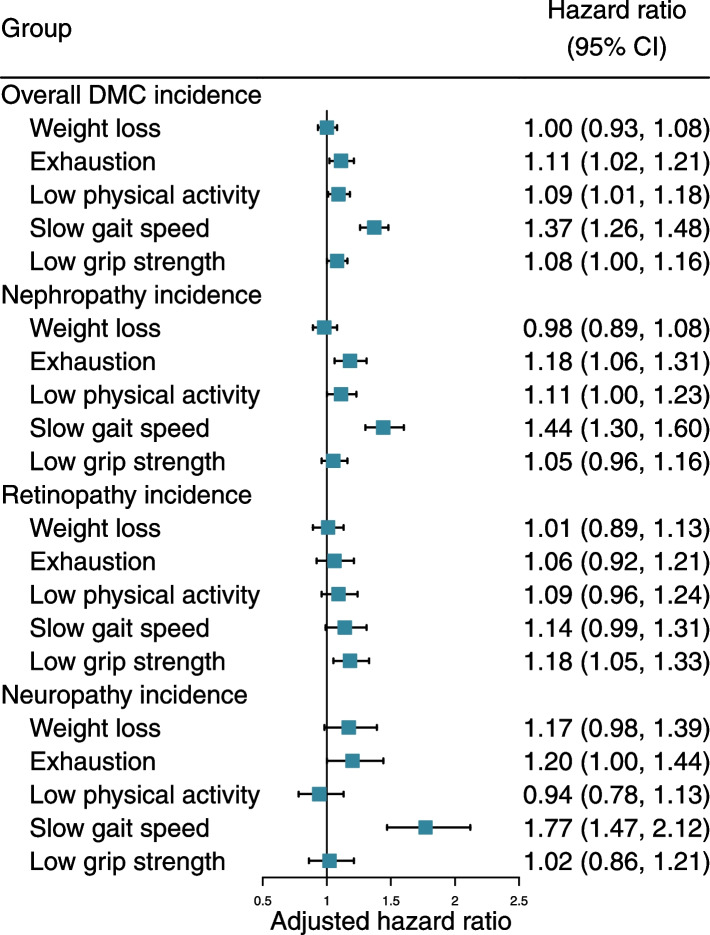
Association between frailty phenotype components and risk of diabetic microvascular complications. Cox proportional hazards models adjusted for age, sex, ethnicity, educational attainment, Townsend deprivation index, annual household income, assessment centers, smoking status, alcohol intake, healthy diet score, BMI, no. of long-term conditions, diabetes duration, HbA1c, diabetes medication use, lipid-lowering treatment, antihypertensive medication use, and aspirin use, and mutually adjusted for other frailty components

The prevalence of weight loss, exhaustion, low physical activity, slow gait speed, and low grip strength in the study population was 26.8%, 17.9%, 20.0%, 19.3%, and 24.9%, respectively (Additional file 1: Table S9). We further analyzed each frailty component per se and the risk of overall DMC and subtypes (Fig. 3 and Additional file 1: Table S10). In the crude model, all five frailty components, except weight loss, were associated with an increased risk of overall DMC and subtypes. After further adjustment for covariates and the five frailty components, the HRs for overall DMC were gradually attenuated, and exhaustion (HR 1.11, 95% CI: 1.02, 1.21), low physical activity (HR 1.09, 95% CI: 1.01, 1.18), slow gait speed (HR 1.37, 95% CI: 1.26, 1.48), and low grip strength (HR 1.08, 95% CI: 1.00, 1.16) were independently associated with risk of over DMC. Exhaustion (HR 1.18, 95% CI: 1.06, 1.31), low physical activity (HR 1.11, 95% CI: 1.00, 1.23), and slow gait speed (HR 1.44, 95% CI: 1.30, 1.60) exhibited a risk association for diabetic nephropathy events. Slow gait speed (HR 1.18, 95% CI: 1.05, 1.33) was associated with higher diabetic retinopathy events. For diabetic neuropathy events, exhaustion (HR 1.20, 95% CI: 1.00, 1.44) and slow gait speed (HR 1.77, 95% CI: 1.47, 2.12) showed a risk association. However, we did not observe a significant association of weight loss with overall DMC and subtypes risk in the mutual adjustment model.

### Secondary analyses

In stratified analyses, the association between frailty phenotype and incident DMC was not significantly modified by all subgroup factors (all *P* for interaction > 0.05) (Additional file 1: Table S11). To minimize the reverse causation, we excluded 353 individuals diagnosed with any microvascular complications or who died within 2 years of follow-up, and the results remained robust (Additional file 1: Table S12). In sensitivity analyses, by sequentially adjusting for lipids, MAP, and eGFR based on model 4, pre-frailty and frailty were still associated with a significantly higher risk of DMC (Additional file 1: Table S13). The associations remained robust in the Fine and Gray competing models (Additional file 1: Table S14). The associations remained robust in sensitivity analyses using an alternative measure of comorbidities, the Charlson Comorbidity Index (Additional file 1: Table S15). In mediation analysis, we found that comorbidities, whether the number of long-term conditions or the Charlson Comorbidity Index, significantly mediated the association between frailty phenotype and incident overall DMC, accounting for 19.8% or 13.1% of the proportion mediated (Additional file 1: Table S16).

## Discussion

In this study of 18,062 middle-aged and older adults with T2D from the UK Biobank, the prevalence of pre-frailty and frailty, based on a widely used Fried frailty phenotype, was 55.8% and 10.4%, respectively. We observed a positive association between frailty score and incident DMC; with each one-point increase in the score, the risk of overall DMC and subtypes event increased by 10 to 20%. Compared with non-frail patients, those with pre-frailty and frailty had a 10 to 52% higher risk of DMC after considering potential confounders. Such associations remained robust in the subtypes of DMC.

The prevalence of frailty and pre-frailty was 10.4% and 55.8% in middle-aged and older adults with T2D, respectively, which is comparable to the rates reported in a previous study from the UK Biobank [34]. However, the prevalence of frailty was lower than that in similar studies that reported 13.0 to 16.3% of diabetes were subjected to frailty [10, 12]. The main reason we speculated was the selection bias of “healthy volunteers” in the UK Biobank, which may have had better health status [35].

To our knowledge, this is the first large study concerning the longitudinal association between frailty phenotype and DMC. Our findings that frailty in T2D was associated with increased risk of DMC events are in line with two prospective studies where frailty was defined as Rockwood’s frailty index and Clinical Frailty Scale [16, 19]. A study by Nguyen et al. [16], where a secondary post hoc analysis of the ADVANCE trial, found that Rockwood’s frailty index was independently associated with increased microvascular events (defined as new or worsening diabetic nephropathy or diabetic retinopathy) (*n* = 11,140, HR: 1.60 [95% CI: 1.42, 1.81]). Another study of 371 diabetes patients aged ≥ 70 years in the Canadian Study of Health and Aging, where frailty was defined using the Clinical Frailty Scale, indicated that frail older adults were 2.62 times (95% CI: 1.36, 5.06) more likely to have a complication of diabetes (diabetic retinopathy, recurrent infections, diabetic nephropathy, and diabetic neuropathy), independent of age, sex, and number of years living with diabetes [19]. However, in a retrospective cohort of 54,505 patients initiating oral antidiabetic drugs from a large US claims and integrated laboratory database, frailty derived from Johns Hopkins-adjusted clinical groups was not associated with new-onset DMC (HR 0.89 [95% CI: 0.70, 1.13]) [20]. Differences in the results may be due to the differences in frailty assessment criteria, study design, and duration of follow-up. In our study, we used the frailty definition described by Fried and colleagues as the clinical manifestations of frailty based on physical phenotypes [21]. They provided a standardized and validated physiologically based definition and clear criteria that are relatively easy and inexpensive to apply and offer a basis for standardized screening for frailty, which now has been one of the most common frailty instruments [8, 9] and the most commonly used measures of frailty in diabetes [12]. We first reported that pre-frailty and frailty based on physical phenotypes were associated with higher DMC risk, and such association remained robust after considering a wealth of covariates, including sociodemographic variables, lifestyles, medical history, medication use, long-term conditions, and glycemic control. In addition, we found consistent results between frailty and diabetic nephropathy, diabetic neuropathy, and diabetic retinopathy, which had not been reported in previous studies that focused only on frailty and overall DMC [16, 19, 20].

In the present study, we found that exhaustion, low physical activity, slow gait speed, and low grip strength were independently associated with an increased risk of overall DMC. Gait speed and handgrip strength are robust predictors of adverse health events in older patients [36, 37]. Previous evidence suggests that physical performance measures are impaired in diabetic patients [38]. Lower grip strength was associated with poor glycemic control in patients with diabetes mellitus (OR: 0.67; 95% CI: 0.47, 0.97) [39]. However, their association with DMC has never been examined, and our study filled this gap and provided new insights into the management of microvascular complications in diabetes.

Although frailty is not currently assessed in routine clinical practice, its importance in diabetes is increasingly recognized in clinical guidelines [40, 41]. Our results support the advocacy of integrating frailty assessment into the routine management of diabetes. In addition to frailty, patients with pre-frailty should be concerned. We found that more than half (55.8%) of T2D have pre-frailty, a higher prevalence than the 49.1% (95% CI: 45.1%, 53.1%) reported in the meta-analysis of community-dwelling older adults with diabetes [10], and they also carried a considerable risk of incident DMC. Pre-frailty is in relation to an increased risk of mortality and cardiovascular events, and imposes greater healthcare expenditure on patients with diabetes [15, 24]. However, few studies have explored the relationship between pre-frailty and incident DMC. More importantly, pre-frailty is a potentially reversible and highly prevalent intermediate state before frailty. Compared to those with no frailty criteria at baseline, individuals with pre-frailty have 2.63 times the risk predisposed to develop into frailty [21]. Our findings fill these evidence gaps that pre-frailty could increase the risk of DMC in patients with diabetes, highlighting the importance of pre-frailty management. Notably, the splines in our results demonstrated a clear positive dose-response correlation between frailty score and overall DMC and three subtypes, implicating that any stage of the frailty progression should be a concern in diabetes management. Regardless of the clinical setting, clinicians are likely to encounter patients with frailty when managing diabetes. In clinical contexts, a nuanced approach (including distinguishing frailty levels and understanding individual patient needs and priorities in the frailty context) may be essential [12]. There is emerging evidence that strategies based on educational, nutritional, and exercise-based interventions seem helpful in delaying or reversing frailty in primary care [42, 43]. As such evidence accumulates, frailty identification, assessment, and management should be part of personalizing treatment for patients with diabetes.

### Potential mechanisms

The development of DMC results from a combination of hyperglycemia-induced endothelial damage, oxidative stress, the production of sorbitol and advanced glycation end-products, pro-inflammation cytokines release, chronic inflammation, protein kinase C activation, and transformation growth factor β upregulation [44, 45]. These metabolic injuries lead to changes in blood flow, endothelial permeability, extravascular protein deposition, and coagulation and induce organ dysfunction [44]. Inflammation and insulin resistance are the precursors of frailty [46] and may also act as the common pathophysiology mechanisms shared by frailty and DMC. Frailty may trigger an inflammatory response, promoting inflammation-mediated insulin resistance and endothelial dysfunction. Besides, loss of muscle mass and strength could lead to metabolic dysregulation resulting in reduced insulin sensitivity, altered oxidative defenses, and decreased mitochondrial function [47], which may promote the occurrence of DMC. Notably, compared with T2D patients free of frailty, those with frailty derive fewer benefits from intensive glucose-lowering and blood pressure-lowering treatments [16], which is known that hyperglycemia and hypertension are common pathophysiological and risk factors for DMC [45].

### Strengths and limitations

Major strengths of the current study included prospective design, long-term follow-up (12.0 years), and high-quality data from the UK Biobank. However, several limitations should be considered. First, some self-reported indicators of frailty phenotype, such as weight loss, exhaustion, and walking speed, were subject to reporting bias, which is usually the main problem in epidemiology. Second, the diagnosis of DMC mainly relied on self-report and the ICD codes, and the incident rates of DMC and its subtypes might be underestimated. The phenomenon might be explained by the “healthy volunteers” bias in the UK Biobank and difficulties for frailty patients to assess medical care and be diagnosed with DMC. Additionally, participants with pre-frailty and frailty may have had undiagnosed DMC at baseline, which might lead to an overestimation of DMC risk for frailty. However, the association was still robust when we excluded participants who developed any DMC or died within 2 years of follow-up. Third, the diagnosis of DMC was identified using cumulative hospital records and death record linkage to national death registries according to the ICD code. Therefore, the potential for misclassification of patients with incident DMC might attenuate findings toward the null, leading to an underestimation of the magnitudes of the true association. Fourth, although we considered a wide range of potential confounders and performed several sensitivity analyses, the residual confounding and potential bias cannot be completely ruled out due to the nature of the observational study. Fifth, as the participants from the UK were predominantly of European ancestry, our findings may not be directly generalizable to other populations, and thus, more research is needed on other ethnic and racial groups.

## Conclusions

In this large prospective cohort of 18,062 diabetes patients, we observed that both pre-frailty and frailty were associated with an increased risk of overall DMC and subtypes, including diabetic nephropathy, diabetic neuropathy, and diabetic retinopathy. The presence of comorbidities partly mediated the association between frailty and the risk of DMC. These findings have important implications for integrating routine clinical assessment and surveillance of frailty into the prevention and management of DMC in diabetes.

## Supplementary Information

**Additional file 1: Figure S1.** Flow of participants in current study. **Table S1.** The definitions for baseline diabetes. **Table S2.** Definition of frailty phenotype and cutoff Points. **Table S3.** Definition of diabetic microvascular complications. **Table S4.** Definition of each component of a healthy diet score. **Table S5.** Definition and list of long-term morbidities. **Table S6.** Definition and list of Charlson Comorbidity Index. **Table S7.** Baseline characteristics of 3678 patients with type 2 diabetes by number of diabetic microvascular complications. **Table S8.** Association between frailty phenotype and risk of diabetic microvascular complications. **Table S9.** Prevalence of frailty phenotype components in percentages. **Table S10.** Association between frailty components and risk of diabetic microvascular complications. **Table S11.** Association between frailty and risk of overall diabetic microvascular complications by subgroups. **Table S12.** Sensitivity analyses of association between frailty phenotype and risk of overall diabetic microvascular complications (exclude individuals diagnosed any microvascular complications within 2 years of follow-up). **Table S13.** Sensitivity analyses of association between frailty phenotype and risk of overall diabetic microvascular complications by sequentially adjusting for lipid profile, MAP, eGFR. **Table S14.** Association between frailty phenotype and risk of overall diabetic microvascular complications using Fine & Gray models for competing risk. **Table S15.** Sensitivity analyses of association between frailty phenotype and risk of diabetic microvascular complications were conducted by using the Charlson Comorbidity Index as an alternative measure of comorbidities**. Table S16.** Mediating effects of multimorbidity on the association of frailty phenotype with risk of diabetic microvascular complications among type 2 diabetes.

## Data Availability

Data from the UK Biobank are available to all researchers upon making an application. This research has been conducted using the UK Biobank Resource under Application 63454.

## Abbreviations

BMI: Body mass index
CI: Confidence interval
DMC: Diabetic microvascular complications
eGFR: Estimated glomerular filtration rate
HbA1c: Glycated hemoglobin A1c
HDL-C: High-density lipoprotein cholesterol
HR: Hazard ratio
ICD: International classification of diseases
IQR: Interquartile range
LDL-C: Low-density lipoprotein cholesterol
MAP: Mean arterial pressure
METs: Metabolic equivalent tasks
SD: Standard deviation
T2D: Type 2 diabetes
TC: Total cholesterol
TG: Triglycerides
