# Supplementary material for: Frailty and risk of microvascular complications in patients with type 2 diabetes: a population-based cohort study
Source: BMC Med. 2022 Dec 8;20:473. doi: 10.1186/s12916-022-02675-9 (PMC9733051; doi:10.1186/s12916-022-02675-9)
Supplement: Supplementary file 1 — Additional file 1: Figure S1. Flow of participants in current study. Table S1. The definitions for baseline diabetes. Table S2. Definition of frailty phenotype and cutoff Points. Table S3. Definition of diabetic microvascular complications. Table S4. Definition of each component of a healthy diet score. Table S5. Definition and list of long-term morbidities. Table S6. Definition and list of Charlson Comorbidity Index. Table S7. Baseline characteristics of 3678 patients with type 2 diabetes by number of diabetic microvascular complications. Table S8. Association between frailty phenotype and risk of diabetic microvascular complications. Table S9. Prevalence of frailty phenotype components in percentages. Table S10. Association between frailty components and risk of diabetic microvascular complications. Table S11. Association between frailty and risk of overall diabetic microvascular complications by subgroups. Table S12. Sensitivity analyses of association between frailty phenotype and risk of overall diabetic microvascular complications (exclude individuals diagnosed any microvascular complications within 2 years of follow-up). Table S13. Sensitivity analyses of association between frailty phenotype and risk of overall diabetic microvascular complications by sequentially adjusting for lipid profile, MAP, eGFR. Table S14. Association between frailty phenotype and risk of overall diabetic microvascular complications using Fine & Gray models for competing risk. Table S15. Sensitivity analyses of association between frailty phenotype and risk of diabetic microvascular complications were conducted by using the Charlson Comorbidity Index as an alternative measure of comorbidities. Table S16. Mediating effects of multimorbidity on the association of frailty phenotype with risk of diabetic microvascular complications among type 2 diabetes. [file 12916_2022_2675_MOESM1_ESM.pdf]

Frailty and Risk of Microvascular Complications in Patients with Type 2 Diabetes: A Population-Based Cohort Study

Supplementary online content

Figure S1. Flow of participants in current study.

Table S1. The definitions for baseline diabetes.

Table S2. Definition of frailty phenotype and cutoff Points.

Table S3. Definition of diabetic microvascular complications.

Table S4. Definition of each component of a healthy diet score.

Table S5. Definition and list of long-term morbidities.

Table S6. Definition and list of Charlson Comorbidity Index.

Table S7. Baseline characteristics of 3678 patients with type 2 diabetes by number of diabetic microvascular complications.

Table S8. Association between frailty phenotype and risk of diabetic microvascular complications.

Table S9. Prevalence of frailty phenotype components in percentages.

Table S10. Association between frailty components and risk of diabetic microvascular complications.

Table S11. Association between frailty and risk of overall diabetic microvascular complications by subgroups.

Table S12. Sensitivity analyses of association between frailty phenotype and risk of overall diabetic microvascular complications (exclude individuals diagnosed any microvascular complications within 2 years of follow-up).

Table S13. Sensitivity analyses of association between frailty phenotype and risk of overall diabetic microvascular complications by sequentially adjusting for lipid profile, MAP, eGFR.

Table S14. Association between frailty phenotype and risk of overall diabetic microvascular complications using Fine & Gray models for competing risk.

Table S15. Sensitivity analyses of association between frailty phenotype and risk of diabetic microvascular complications were conducted by using the Charlson comorbidity index as an alternative measure of comorbidities**.**

Table S16. Mediating effects of multimorbidity on the association of frailty phenotype with risk of diabetic microvascular complications among type 2 diabetes.

Fig. S1. Flow of participants in current study

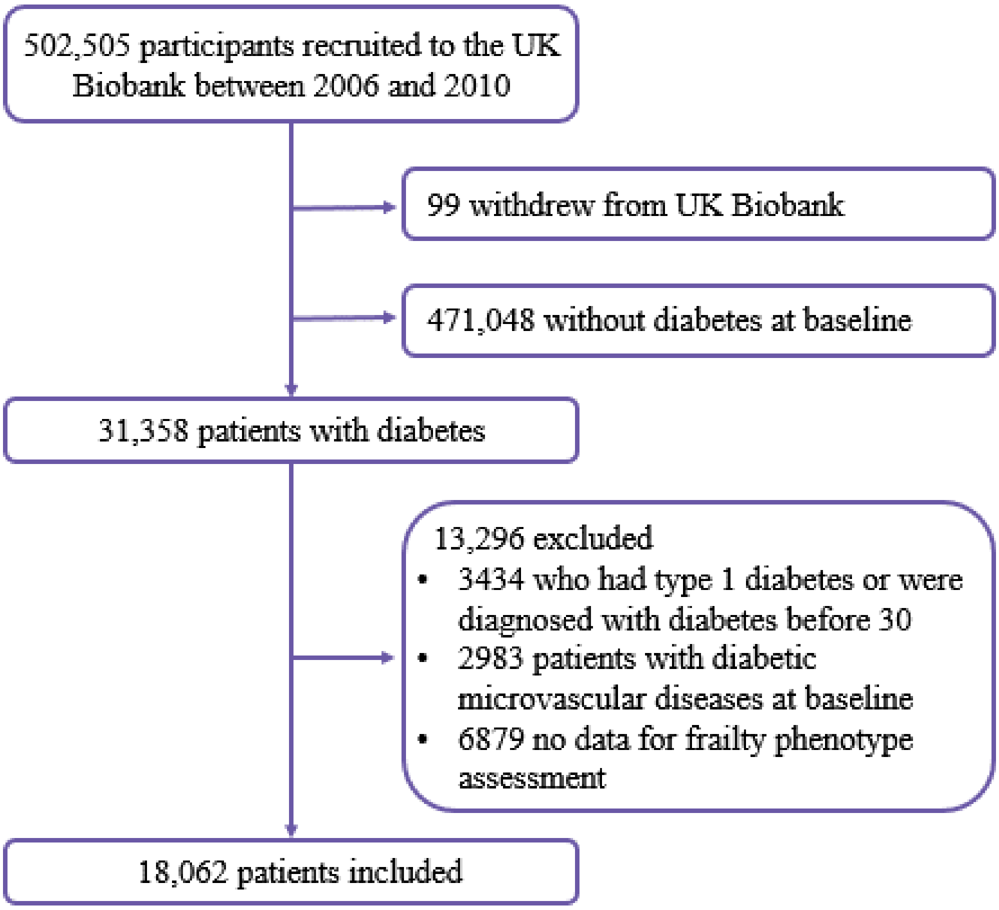

Table S1. The definitions for baseline diabetes [a].

	ICD-9	ICD-10	Self-reported field code	Examination
**Diabetes at baseline**	250	E11, E12, E13, E14	2443, 2976, 6153, 6177, 20002, 20003	30750

[a] Variable definitions constructed using ICD-9, ICD-10 as well as self-reported data fields with choice-, disease- or procedure-specific codes between brackets are shown.

Abbreviations: ICD, International Classification of Diseases

Table S2. Definition of frailty phenotype and cutoff Points.

Components	Criteria	Field IDs
**Weight loss**	Self-reported: "Compared with one year ago, has your weight changed?"<br>Response: yes, lost weight = 1; other = 0;<br>Do not know/Prefer not to answer = missing data.	2306
**Exhaustion**	Self-reported: "Over the past two weeks, how often have you felt tired or had little energy?"<br>Response: more than half the days or nearly every day = 1; other = 0;<br>Do not know/Prefer not to answer = missing data.	2080
**Low physical activity**	Quintiles of sex- and age-specific levels of total MET minutes per week derived from IPAQ.<br>The lowest 20% of total MET minutes per week = 1; other = 0;<br>No response/ Prefer not to answer = missing data.	31, 21022, 22037, 22038, 22039
**Slow gait speed**	Self-reported: "How would you describe your usual walking pace?"<br>Response: slow = 1; other = 0;<br>Do not know/Prefer not to answer = missing data.	924
**Low grip strength**	Measured grip strength expressed in kg by sex- and BMI- adjusted cut-off points.<br>Cut-off points:<br>Men<br>If BMI ≤24.0 kg/m^2 & grip strength ≤29 kg<br>If BMI 24.1 to 26.0 kg/m^2 & grip strength ≤30 kg<br>If BMI 26.1 to 28.0 kg/m^2 & grip strength ≤30 kg<br>If BMI >28.0 kg/m^2 & grip strength ≤32 kg<br>Women<br>If BMI ≤23.0 kg/m^2 & grip strength ≤17 kg<br>If BMI 23.1 to 26.0 kg/m^2 & grip strength ≤17.3 kg<br>If BMI 26.1 to 29.0 kg/m^2 & grip strength ≤18 kg<br>If BMI >29.0 kg/m^2 & grip strength ≤21 kg	31, 21001, 46, 47

Table S3. Definition of diabetic microvascular complications [a].

	ICD-9	ICD-10	Self-reported field IDs
**Nephropathy**	2503	E112, E122, E132, E142, N083, N180, N181, N182, N183, N184, N185, N186, N187, N188, N189	20002 (1607)
**Retinopathy**	2504	E113, E123, E133, E143, H280, H360	6148, 20002 (1276)
**Neuropathy**	2505	E114, E124, E134, E144, G590, G629, G632, G990	20002 (1468)

[a] Variable definitions constructed using ICD-9, ICD-10 as well as self-reported data fields with choice-, disease- or procedure-specific codes between brackets are shown.

Abbreviations: ICD, International Classification of Diseases

Table S4. Definition of each component of a healthy diet score [a].

	Goal (1 point)	One serving equals to	Field IDs
**Fruits**	≥ 3 servings/day	1 piece of fresh fruit<br>5 pieces of dried fruit	1309, 1319
**Vegetables (excluding potatoes)**	≥ 3 servings/day	3 heaped tablespoons	1289, 1299
**Whole grains**	≥ 3 servings/day	1 slice of whole-grain bread<br>1 cup of whole-grain cereal	1438, 1448, 1458, 1468
**Vegetable oil**	≥ 2 servings/day	Vegetable oil based spread (Flora Pro-Active/Benecol, Soft (tub) margarine, Olive oil based spread, or Polyunsaturated/sunflower oil based spread) in combination with eating at least 2 slices of bread	1428, 2654, 1438
**Fish**	≥ 2 servings/week	Once/week	1329, 1339
**Dairy**	≥ 2 servings/day	1 cup/day if consumption any type of milk<br>1 piece of cheese	1408, 1418
**Refined grains**	≤ 2 servings/day	1 slice of bread or 1 bowl of cereal	1438, 1448, 1458, 1468
**Unprocessed meats**	≤ 2 servings/week	Once/week (including poultry, beef, lamb, and pork)	1359, 1369, 1379, 1389
**Processed meats**	≤ 1 servings/week	Once/week	1349
**Sugar-sweetened beverages**	Don't drink	Only 0 serving was possible here	6144

[a] Field IDs and serving sizes used per diet component in UK Biobank with available data from the general baseline questionnaire. If participants achieved the intake goal they were considered to have an adequate intake of the diet component. Adequate intake of at least half of all diet components was considered as an ideal diet, less than half was considered a poor diet.

Table S5. Definition and list of long-term morbidities.

	Morbidity grouping*	Conditions included	Code
**1**	**Hypertension**	Hypertension	1065
		Essential hypertension	1072
**2**	**Coronary heart disease**	Heart attack/MI	1075
		Angina	1074
**3**	**Diabetes**	Diabetic nephropathy	1607
		Diabetic neuropathy/ulcers	1468
		Diabetes	1220
		Type 1 diabetes	1222
		Type 2 diabetes	1223
		Diabetic eye disease	1276
**4**	**Stroke/TIA**	Stroke	1081
		TIA	1082
		Subarachnoid haemorrhage	1086
		Brain haemorrhage	1491
		Ischaemic stroke	1583
**5**	**Atrial fibrillation**	Atrial fibrillation	1471
**6**	**Heart failure**	Cardiomyopathy	1079
		Hypertrophic cardiomyopathy	1588
		Heart failure/pulmonary oedema	1076
**7**	**Peripheral vascular disease**	Peripheral vascular disease	1067
		Leg claudication/intermittent claudication	1087
**8**	**COPD**	COPD/Chronic obstructive pulmonary disease	1112
		Emphysema/Chronic bronchitis	1113
		Emphysema	1472
**9**	**Asthma**	Asthma	1111
**10**	**Bronchiectasis**	Bronchiectasis	1114
**11**	**Cancer***	“yes”/”no” to “have you ever had cancer?”	
**12**	**Dyspepsia**	Gastro-oesophageal reflux (GORD)	1138
		Oesophagitis/Barrett’s oesophagus	1139
		Gastric stomach ulcers	1142
		Gastric erosions/gastritis	1143
		Duodenal ulcer	1457
		Dyspepsia/indigestion	1510
		Hiatus hernia	1474
		Helicobacter pylori	1442

**13**	**Diverticular disease**	Diverticular disease/diverticulitis	1458
**14**	**Irritable bowel syndrome**	Irritable bowel syndrome	1154
**15**	**Chronic liver disease**	Oesophageal varices	1141
		Non infective hepatitis	1157
		Liver failure/cirrhosis	1158
		Primary biliary cirrhosis	1506
**16**	**Inflammatory bowel disease**	Inflammatory bowel disease	1461
		Crohn’s disease	1462
		Ulcerative colitis	1463
**17**	**Constipation**	Constipation	1599
**18**	**Viral hepatitis**	Hepatitis B	1579
		Hepatitis C	1580
		Hepatitis D	1581
**19**	**Depression**	Depression	1286
		Postnatal depression	1531
**20**	**Anxiety**	Anxiety/panic attacks	1287
		Nervous breakdown	1288
		Post-traumatic stress disorder	1469
		Obsessive compulsive disorder	1615
		Stress	1614
		Insomnia	1616
		Psychological/psychiatric problem	1243
**21**	**Schizophrenia/Bipolar affective disorder**	Scizophrenia	1289
**21**	**Bipolar**	Mania	1291
		Bipolar disorder	1291
		Manic depression	1291
**22**	**Connective tissue diseases**	Myositis/myopathy	1322
		Systemic lupus erythematosus/SLE	1381
		Connective tissue disorder	1373
		Sjogren’s syndrome.sicca syndrome	1382
		Dermatopolymyositis	1383
		Scloeroderma/systemic sclerosis	1384
		Rheumatoid arthritis	1464
		Psoriatic arthropathy	1477
		Dermatomyositis	1480
		Polymyositis	1481
		Polymyalgia rheumatica	1377
**23**	**Painful conditions**	Back pain	1534
		Joint pain	1537

		Headaches (not migraine)	1436
		Sciatica	1476
		Plantar fasciitis	1540
		Carpal tunnel syndrome	1541
		Fibromyalgia	1542
		Arthritis	1538
		Shingles	1573
		Disc problem	1532
		Prolapsed disc/slipped disc	1312
		Spine arthritis/spondylitis	1311
		Ankylosing spondylitis	1313
		Back problem	1294
		Osteoarthritis	1465
		Gout	1466
		Cervical spondylosis	1478
		Trigeminal neuralgia	1523
		Disc degeneration	1533
		Trapped nerve/compressed nerve	1257
**24**	**Osteoporosis**	Osteoporosis	1309
**25**	**Thyroid disorders**	Thyroid problem (not cancer)	1224
		Hyperthroidism/thyrotoxicosis	1225
		Hypothyroidism/myxoedema	1226
		Grave’s disease	1522
		Thyroid goitre	1610
		Thyroitits	1428
**26**	**Alcohol problems**	Alcohol dependency	1408
		Alcoholic liver disease/alcoholic cirrhosis	1604
**27**	**Chronic kidney disease**	Polycystic kidney	1427
		Diabetic nephropathy	1607
		Renal/kidney failure	1192
		Renal failure requiring dialysis	1193
		Renal failure not requiring dialysis	1194
		Kidney nephropathy	1519
		Immunoglobulin A (IgA) nephropathy	1520
**28**	**Prostate disorders**	Prostate problem (not cancer)	1207
		Enlarged prostate	1396
		Benign prostatic hypertrophy	1516
**29**	**Glaucoma**	Glaucoma	1277
**30**	**Epilepsy**	Epilepsy	1264

**31**	**Dementia**	Dementia/Alzheimer/cognitive impairment	1263
**32**	**Psoriasis or eczema**	Eczema/dermatitis	1452
		Psoriasis	1453
**33**	**Migraine**	Migraine	1265
**34**	**Chronic sinusitis**	Chronic sinusitis	1416
**35**	**Anorexia or bulimia**	Anorexia, bulimia/other eating disorder	1470
**36**	**Parkinson's disease**	Parkinson's disease	1262
**37**	**Multiple sclerosis**	Multiple sclerosis	1261
**38**	**Chronic fatigue syndrome**	Chronic fatigue syndrome	1482
**39**	**Endometriosis**	Endometriosis	1402
**40**	**Meniere disease**	Meniere disease	1421
**41**	**Pernicious anaemia**	Pernicious anaemia	1331
**42**	**Polycystic ovaries**	Polycystic ovaries	1350

*Self-report lifetime diagnosis by doctor recorded by interview (UK Biobank data field ID: 20002), except cancer diagnosis which was reported by touch-screen questionnaire. The list of disease groupings was based on Barnett et al (2012). In the present study, we did not include patients with diabetes at baseline.

Table S6. Definition and list of Charlson Comorbidity Index [a].

	Comorbidities	Weight	ICD-9	ICD-10	Self-reported fields and doctor-diagnosis fields
1	Congestive heart failure	2	410, 4109, 411, 4119, 412, 4129, 413, 4139, 414, 4140, 4141, 4148, 4149	I20, I200, I201, I208, I209, I21, I210, I211, I212, I213, I214, I219, I21X, I22, I220, I221, I228, I229, I23, I230, I231, I232, I233, I234, I235, I236, I238, I24, I240, I241, I248, I249, I25, I250, I251, I252, I253, I254, I255, I256, I258, I259	6150<br>20004 (1069, 1070, 1071, 1095, 1523)<br>20002 (1075)<br>41272
2	Dementia	2	3310, 3311	F00, F000, F001, F002, F009, G30, G300, G301, G308, G309, F01, F010, F011, F012, F013, F018, F019, I673, F020, G310	20002 (1263)
3	Chronic pulmonary disease	1	4168, 4169, 4909, 4910, 4911, 4912, 4918, 4919, 4929, 4949, 4950, 4951, 4952, 4953, 4954, 4955, 4956, 4957, 4958, 4959, 4969, 5009, 5019, 5029, 5039, 5049, 5059	I278, I279, J40, J410, J411, J418, J42, J430, J431, J432, J438, J439, J440, J441, J448, J449, J450, J451, J458, J459, J46, J47, J60, J61, J620, J628, J630, J631, J632, J633, J634, J635, J638, J64, J65, J660, J661, J662, J668, J670, J671, J672, J673, J674, J675, J676, J677, J678, J679, J684, J701, J703	20002 (1112,1113,1114,1115,1472,1121)<br>6152, 3992
4	Rheumatologic disease	1	4465, 7100, 7101, 7102, 7103, 7104, 7140, 71400, 71401, 71402, 71403, 71404, 71405, 71406, 71407, 71408, 71409, 7141, 71410, 71411, 71412, 71413, 71414, 71415, 71416, 71417, 71418, 71419, 7142, 71420, 71421, 71422, 71423, 71424, 71425, 71426, 71427,	M0500, M0501, M0502, M0503, M0504, M0505, M0506, M0507, M0508, M0509, M0510, M0511, M0512, M0513, M0514, M0515, M0516, M0517, M0518, M0519, M0520, M0521, M0522, M0523, M0524, M0525, M0526, M0527, M0528, M0529, M0530, M0531, M0532, M0533, M0534, M0535, M0536, M0537, M0538, M0539, M0580, M0581,	20002 (1377,1464)

			71428, 71429,72590, 72591, 72592, 72593, 72594, 72595, 72596, 72597, 72598, 72599	M0582, M0583, M0584, M0585, M0586, M0587, M0588, M0589, M0590, M0591, M0592, M0593, M0594, M0595, M0596, M0597, M0598, M0599, M0600, M0601, M0602, M0603, M0604, M0605, M0606, M0607, M0608, M0609, M0610, M0611, M0612, M0613, M0614, M0615, M0616, M0617, M0618, M0619, M0620, M0621, M0622, M0623, M0624, M0625, M0626, M0627, M0628, M0629, M0630, M0631, M0632, M0633, M0634, M0635, M0636, M0637, M0638, M0639, M0640, M0641, M0642, M0643, M0644, M0645, M0646, M0647, M0648, M0649, M0680, M0681, M0682, M0683, M0684, M0685, M0686, M0687, M0688, M0689, M0690, M0691, M0692, M0693, M0694, M0695, M0696, M0697, M0698, M0699, M320, M321, M328, M3290, M330, M331, M332, M339, M315, M351, M353, M360	
5	Mild liver disease	2	702, 703, 704, 705, 706, 709, 5709, 5710, 5711, 5712, 5713, 5714, 5716, 5718, 5719, 5733, 5734, 5738, 5739, V427	B180, B181, B182, B188, B189, K700, K701, K702, K703, K709, K713, K714, K715, K717, K730, K731, K732, K738, K739, K740, K741, K742, K743, K744, K745, K746, K760, K762, K763, K764, K768, K769, Z944	20002 (1136, 1155, 1156, 1157, 1506, 1578, 1579, 1580, 1581, 1582, 1604)
6	Diabetes with chronic complications	1	2504, 2505, 2506, 2507	E102, E103, E104, E105, E107, E112, E113, E114, E115, E117, E122, E123, E124, E125, E127, E132, E133, E134, E135, E137, E142, E143, E144, E145, E147	6148,<br>20002 (1276)

7	Hemiplegia or paraplegia	2	3341, 3420, 3421, 3429, 34300, 34301, 3431, 3432, 3433, 3434, 3438, 3439, 3440, 3441, 3442, 3443, 3444, 3445, 3446, 3449	G041, G114, G801, G802, G810, G811, G819, G820, G821, G822, G823, G824, G825, G830, G831, G832, G833, G834, G839	20002 (1252)
8	Renal disease	1	4030, 4031, 4039, 4040, 4041, 4049, 5820, 5821, 5822, 5824, 5828, 5829, 5830, 5831, 5832, 5834, 5836, 5837, 5859, 5869, 5880, V420, V451, V560, V568	I120, I131, N032, N033, N034, N035, N036, N037, N052, N053, N054, N055, N056, N057, N180, N181, N182, N183, N184, N185, N188, N189, N19, N250, Z490, Z491, Z492, Z940, Z992	20002 (1405,1192,1193,1194) 131291
9	Any malignancy, including leukemia and lymphoma	2	1400, 1401, 1403, 1404, 1405, 1406, 1408, 1409, 1410, 1411, 1412, 1413, 1414, 1415, 1416, 1418, 1419, 1420, 1421, 1422, 1428, 1429, 1430, 1431, 1438, 1439, 1440, 1441, 1448, 1449, 1450, 1451, 1452, 1453, 1454, 1455, 1456, 1458, 1459, 1460, 1461, 1462, 1463, 1464, 1465, 1466, 1467, 1468, 1469, 1470, 1471, 1472, 1473, 1478, 1479, 1480, 1481, 1482, 1483, 1488, 1489, 1490, 1490, 1491, 1491, 1492, 1493, 1494, 1495, 1498, 1499, 1499, 1500, 1501, 1502, 1503, 1504, 1505, 1508, 1509, 1510, 1510, 1511, 1511, 1512, 1512, 1513, 1513, 1514, 1514, 1515, 1515, 1516, 1518, 1519, 1519, 1520, 1520, 1521, 1521, 1522, 1522, 1523, 1523, 1524, 1525, 1528, 1529, 1529, 1530, 1531, 1532, 1533, 1534, 1535, 1536, 1537, 1538, 1539, 1540, 1541, 1542, 1543, 1548, 1550, 1551, 1552, 1560, 1561, 1562, 1568, 1569, 1570,	C000, C001, C002, C003, C004, C005, C006, C008, C009, C01, C020, C021, C022, C023, C024, C028, C029, C030, C031, C039, C040, C041, C048, C049, C050, C051, C052, C058, C059, C060, C061, C062, C068, C069, C07, C080, C081, C088, C089, C090, C091, C098, C099, C100, C101, C102, C103, C104, C108, C109, C110, C111, C112, C113, C118, C119, C12, C130, C131, C132, C138, C139, C140, C142, C148, C150, C151, C152, C153, C154, C155, C158, C159, C160, C161, C162, C163, C164, C165, C166, C168, C169, C170, C171, C172, C173, C178, C179, C180, C181, C182, C183, C184, C185, C186, C187, C188, C189, C19, C20, C210, C211, C212, C218, C220, C221, C222, C223, C224, C227, C229, C23, C240, C241, C248, C249, C250, C251, C252, C253, C254, C257, C258, C259, C260, C261, C268, C269, C300, C301, C310, C311, C312, C313, C318, C319, C320, C321,	20001 (1001, 1002, 1004, 1005, 1006, 1007, 1008, 1009, 1010, 1011, 1012, 1015, 1016, 1017, 1018, 1019, 1020, 1021, 1022, 1023, 1024, 1025, 1026, 1027, 1028, 1029, 1030, 1031, 1032, 1033, 1034, 1035, 1036, 1037, 1038, 1039, 1040, 1041, 1042, 1043, 1044, 1045, 1046, 1047, 1048, 1050, 1051, 1052, 1053, 1055, 1056, 1058, 1061, 1062, 1063, 1064, 1065, 1066, 1067, 1068, 1070, 1072, 1073, 1074, 1075, 1076, 1077, 1078, 1079, 1080, 1081, 1082, 1084, 1085, 1086, 1087, 1088, 99999)

			1570, 1571, 1571, 1572, 1572, 1573, 1573,	C322, C323, C328, C329, C33, C340, C341, C342,	
			1574, 1574, 1575, 1578, 1579, 1579, 1580,	C343, C348, C349, C37, C380, C381, C382, C383,	
			1580, 1581, 1582, 1583, 1584, 1585, 1588,	C384, C388, C390, C398, C399, C400, C401,	
			1589, 1589, 1590, 1590, 1591, 1591, 1592,	C402, C403, C408, C409, C410, C411, C412,	
			1593, 1594, 1595, 1598, 1599, 1599, 1600,	C413, C414, C418, C419, C430, C431, C432,	
			1600, 1601, 1601, 1602, 1602, 1603, 1603,	C433, C434, C435, C436, C437, C438, C439,	
			1604, 1604, 1605, 1605, 1608, 1609, 1609,	C450, C451, C452, C457, C459, C460, C461,	
			1610, 1610, 1611, 1611, 1612, 1612, 1613,	C462, C463, C467, C468, C469, C470, C471,	
			1613, 1614, 1615, 1618, 1619, 1619, 1620,	C472, C473, C474, C475, C476, C478, C479,	
			1622, 1623, 1624, 1625, 1628, 1629, 1630,	C480, C481, C482, C488, C490, C491, C492,	
			1630, 1631, 1631, 1632, 1633, 1634, 1635,	C493, C494, C495, C496, C498, C499, C500,	
			1638, 1639, 1639, 1640, 1640, 1641, 1641,	C501, C502, C503, C504, C505, C506, C508,	
			1642, 1642, 1643, 1643, 1644, 1645, 1648,	C509, C510, C511, C512, C518, C519, C52, C530,	
			1649, 1649, 1650, 1658, 1659, 1690, 1690,	C531, C538, C539, C540, C541, C542, C543,	
			1691, 1691, 1692, 1692, 1693, 1693, 1694,	C548, C549, C55, C56, C570, C571, C572, C573,	
			1695, 1699, 1699, 1700, 1700, 1701, 1701,	C574, C577, C578, C579, C58, C600, C601, C602,	
			1702, 1702, 1703, 1703, 1704, 1704, 1705,	C608, C609, C61, C620, C621, C629, C630, C631,	
			1705, 1706, 1707, 1708, 1709, 1709, 1710,	C632, C637, C638, C639, C64, C65, C66, C670,	
			1710, 1711, 1712, 1712, 1713, 1713, 1714,	C671, C672, C673, C674, C675, C676, C677,	
			1714, 1715, 1715, 1716, 1717, 1718, 1719,	C678, C679, C680, C681, C688, C689, C690,	
			1719, 1720, 1720, 1721, 1721, 1722, 1722,	C691, C692, C693, C694, C695, C696, C698,	
			1723, 1723, 1724, 1724, 1725, 1725, 1726,	C699, C700, C701, C709, C710, C711, C712,	
			1727, 1728, 1729, 1729, 1740, 1740, 1741,	C713, C714, C715, C716, C717, C718, C719,	
			1741, 1742, 1742, 1743, 1743, 1744, 1744,	C720, C721, C722, C723, C724, C725, C728,	
			1745, 1745, 1746, 1748, 1749, 1749, 1750,	C729, C73, C740, C741, C749, C750, C751, C752,	
			1751, 1752, 1753, 1754, 1755, 1759, 1759,	C753, C754, C755, C758, C759, C760, C761,	

			1770, 1771, 1772, 1773, 1774, 1775, 1779, 1780, 1781, 1782, 1783, 1784, 1785, 1789, 1799, 1800, 1800, 1801, 1801, 1802, 1803, 1804, 1805, 1808, 1809, 1809, 1819, 1820, 1821, 1828, 1830, 1832, 1833, 1834, 1835, 1838, 1839, 1840, 1841, 1842, 1843, 1844, 1848, 1849, 1859, 1860, 1869, 1871, 1872, 1873, 1874, 1875, 1876, 1877, 1878, 1879, 1880, 1880, 1881, 1881, 1882, 1882, 1883, 1883, 1884, 1884, 1885, 1885, 1886, 1887, 1888, 1889, 1889, 1890, 1890, 1891, 1891, 1892, 1892, 1893, 1893, 1894, 1894, 1895, 1898, 1899, 1899, 1900, 1901, 1902, 1903, 1904, 1905, 1906, 1907, 1908, 1909, 1910, 1911, 1912, 1913, 1914, 1915, 1916, 1917, 1918, 1919, 1920, 1921, 1922, 1923, 1928, 1929, 1939, 1940, 1941, 1943, 1944, 1945, 1946, 1948, 1949, 1950, 1951, 1952, 1953, 1954, 1955, 1958, 2000, 2001, 2002, 2008, 2010, 2011, 2012, 2014, 2015, 2016, 2017, 2019, 2020, 2021, 2022, 2023, 2024, 2025, 2026, 2028, 2029, 2030, 2031, 2038, 2040, 2041, 2042, 2048, 2049, 2050, 2051, 2052, 2053, 2058, 2059, 2060, 2061, 2062, 2068, 2069, 2070, 2071, 2072, 2078, 2080, 2081, 2082, 2088, 2089, 2386	C762, C763, C764, C765, C767, C768, C810, C811, C812, C813, C814, C817, C819, C820, C821, C822, C823, C824, C825, C826, C827, C829, C830, C831, C832, C833, C834, C835, C836, C837, C838, C839, C840, C841, C842, C843, C844, C845, C846, C847, C848, C849, C850, C851, C852, C857, C859, C880, C881, C882, C883, C884, C887, C889, C900, C901, C902, C903, C910, C911, C912, C913, C914, C915, C916, C917, C918, C919, C920, C921, C922, C923, C924, C925, C926, C927, C928, C929, C930, C931, C932, C933, C937, C939, C940, C941, C942, C943, C944, C945, C946, C947, C950, C951, C952, C957, C959, C960, C961, C962, C963, C964, C965, C966, C967, C968, C969, C97	

10	Moderate or severe liver disease	4	4560, 4561, 4562, 5722, 5723, 5724, 5728	I850, I859, I864, I982, K704, K711, K721, K729, K765, K766, K767	20002 (1141,1158)
11	Metastatic solid tumor	6	1960, 1961, 1962, 1963, 1965, 1966, 1968, 1969, 1970, 1971, 1972, 1973, 1974, 1975, 1976, 1977, 1978, 1980, 1981, 1982, 1983, 1984, 1985, 1986, 1987, 1988, 1990, 1991	C770, C771, C772, C773, C774, C775, C778, C779, C780, C781, C782, C783, C784, C785, C786, C787, C788, C790, C791, C792, C793, C794, C795, C796, C797, C798, C799, C800, C809	20001 (1071)
12	AIDS/HIV	4	042, 043, 044, 27917	B200, B201, B202, B203, B204, B205, B206, B207, B208, B209, B210, B211, B212, B213, B217, B218, B219, B220, B221, B222, B227, B24	20002 (1439)

[a] The Charlson comorbidity index consists of 12 comorbidities and a total index ranging from 0 to 24.

Table S7. Baseline characteristics of 3678 patients with type 2 diabetes by number of diabetic microvascular complications [a].

Variables	Overall	Number of diabetic microvascular complications			*P* value
		One	Two	Three	
Number of participants	3678	3036	556	86	
Age, mean (SD), years	61.7 (6.2)	61.7 (6.1)	61.8 (6.4)	59.9 (6.7)	0.019
BMI, mean (SD), kg/m^2	32.2 (5.8)	32.1 (5.8)	32.7 (5.8)	32.6 (5.9)	0.039
Male, n (%)	2382 (64.8%)	1939 (63.9%)	380 (68.3%)	63 (73.3%)	0.032
White ethnicity, n (%)	3299 (89.7%)	2724 (89.7%)	499 (89.7%)	76 (88.4%)	0.206
Townsend deprivation index, median (IQR)	-1.3 (-3.2 to 1.9)	-1.4 (-3.3 to 1.8)	-0.9 (-3.0 to 2.1)	-0.6 (-2.6 to 2.3)	0.013
Education, n (%)					0.054
College or university	843 (22.9%)	706 (23.3%)	119 (21.4%)	18 (20.9%)	
Vocational	590 (16.0%)	467 (15.4%)	113 (20.3%)	10 (11.6%)	
Upper secondary	322 (8.8%)	266 (8.8%)	45 (8.1%)	11 (12.8%)	
Lower secondary	866 (23.5%)	733 (24.1%)	112 (20.1%)	21 (24.4%)	
Others	1019 (27.7%)	829 (27.3%)	165 (29.7%)	25 (29.1%)	
Unknown	38 (1.0%)	35 (1.2%)	2 (0.4%)	1 (1.2%)	
Annual Household income, n (%), £					0.246
<18,000	1284 (34.9%)	1048 (34.5%)	202 (36.3%)	34 (39.5%)	
18,000-30,999	933 (25.4%)	777 (25.6%)	135 (24.3%)	21 (24.4%)	
31,000-51,999	566 (15.4%)	470 (15.5%)	82 (14.7%)	14 (16.3%)	
52,000-100,000	320 (8.7%)	265 (8.7%)	54 (9.7%)	1 (1.2%)	
>100,000	46 (1.3%)	43 (1.4%)	2 (0.4%)	1 (1.2%)	
Unknown	529 (14.4%)	433 (14.3%)	81 (14.6%)	15 (17.4%)	
Smoking status, n (%)					0.266
Never	1456 (39.6%)	1228 (40.4%)	194 (34.9%)	34 (39.5%)	
Former	1794 (48.8%)	1464 (48.2%)	289 (52.0%)	41 (47.7%)	
Current	417 (11.3%)	336 (11.1%)	70 (12.6%)	11 (12.8%)	

Unknown	11 (0.3%)	8 (0.3%)	3 (0.5%)	0 (0.0%)	
Alcohol intake, median (IQR), g/day	8.5 (0.8 to 21.3)	8.5 (0.8 to 21.3)	8.5 (0.4 to 20.5)	6.2 (0.1 to 17.9)	0.360
Healthy diet score, mean (SD)	3.0 (2.0 to 4.0)	3.0 (2.0 to 4.0)	3.0 (2.0 to 4.0)	3.0 (2.0 to 4.0)	0.962
Physical activity, median (IQR), MET-h/week	21.6 (7.9 to 47.5)	21.5 (8.2 to 48.3)	22.2 (7.8 to 44.7)	18.6 (5.8 to 51.2)	0.585
Diabetes duration, n (%), years					<0.001
<1	605 (16.4%)	539 (17.8%)	60 (10.8%)	6 (7.0%)	
1–4.9	1131 (30.8%)	970 (31.9%)	142 (25.5%)	19 (22.1%)	
5–9.9	1043 (28.4%)	844 (27.8%)	171 (30.8%)	28 (32.6%)	
≥10	899 (24.4%)	683 (22.5%)	183 (32.9%)	33 (38.4%)	
Diabetes medication use, n (%)					<0.001
None	1178 (32.0%)	1048 (34.5%)	121 (21.8%)	9 (10.5%)	
Only oral medication	1904 (51.8%)	1560 (51.4%)	302 (54.3%)	42 (48.8%)	
Only insulin	183 (5.0%)	130 (4.3%)	38 (6.8%)	15 (17.4%)	
Insulin and oral medication	413 (11.2%)	298 (9.8%)	95 (17.1%)	20 (23.3%)	
HbA1c, n (%), mmol/mol					<0.001
< 53.0	1965 (53.4%)	1685 (55.5%)	249 (44.8%)	31 (36.0%)	
≥53.0	1471 (40.0%)	1148 (37.8%)	272 (48.9%)	51 (59.3%)	
Missing	242 (6.6%)	203 (6.7%)	35 (6.3%)	4 (4.7%)	
Antihypertensive medication use, n (%)	2570 (69.9%)	2085 (68.7%)	419 (75.4%)	66 (76.7%)	0.003
Lipid-lowering treatment, n (%)	2901 (78.9%)	2366 (77.9%)	463 (83.3%)	72 (83.7%)	0.010
Aspirin use, n (%)	1931 (52.5%)	1561 (51.4%)	319 (57.4%)	51 (59.3%)	0.016
No. of long-term conditions, n (%)					0.271
0	387 (10.5%)	338 (11.1%)	43 (7.7%)	6 (7.0%)	
1	1042 (28.3%)	842 (27.7%)	172 (30.9%)	28 (32.6%)	
2	1037 (28.2%)	867 (28.6%)	147 (26.4%)	23 (26.7%)	
3	688 (18.7%)	557 (18.3%)	112 (20.1%)	19 (22.1%)	
4	329 (8.9%)	270 (8.9%)	51 (9.2%)	8 (9.3%)	
≥5	195 (5.3%)	162 (5.3%)	31 (5.6%)	2 (2.3%)	

[a] Abbreviations: BMI, body mass index; HbA1c, glycated hemoglobin A1c; MET, metabolic equivalent; IQR, interquartile range; SD, standard deviation.

Table S8. Association between frailty phenotype and risk of diabetic microvascular complications [a].

	Frailty phenotype			Per score increment	*P* trend
	Non-frail	Pre-frail	Frail		
N	6101	10073	1888		
**Overall microvascular complications**					
Cases/person-years	1067/69322	2031/110690	580/18701		
Incident rate per 1000 person-years	15.39	18.35	31.01		
Model 1	1.00 (reference)	1.21 (1.12, 1.30)	2.08 (1.88, 2.31)	1.25 (1.21, 1.28)	<0.0001
Model 2	1.00 (reference)	1.14 (1.06, 1.23)	1.81 (1.62, 2.01)	1.19 (1.16, 1.23)	<0.0001
Model 3	1.00 (reference)	1.10 (1.02, 1.19)	1.61 (1.44, 1.79)	1.15 (1.12, 1.19)	<0.0001
Model 4	1.00 (reference)	1.10 (1.02, 1.18)	1.52 (1.36, 1.69)	1.13 (1.10, 1.17)	<0.0001
**Nephropathy**					
Cases/person-years	588/71261	1235/114293	390/19609		
Incident rate per 1000 person-years	8.25	10.81	19.89		
Model 1	1.00 (reference)	1.34 (1.21, 1.48)	2.46 (2.15, 2.80)	1.32 (1.27, 1.37)	<0.0001
Model 2	1.00 (reference)	1.23 (1.11, 1.36)	2.00 (1.75, 2.30)	1.24 (1.19, 1.29)	<0.0001
Model 3	1.00 (reference)	1.16 (1.05, 1.28)	1.67 (1.45, 1.92)	1.17 (1.13, 1.22)	<0.0001
Model 4	1.00 (reference)	1.15 (1.04, 1.27)	1.59 (1.38, 1.83)	1.16 (1.11, 1.20)	<0.0001
**Retinopathy**					
Cases/person-years	481/71321	847/115116	192/20359		
Incident rate per 1000 person-years	6.74	7.36	9.43		
Model 1	1.00 (reference)	1.10 (0.98, 1.23)	1.45 (1.22, 1.72)	1.13 (1.08, 1.18)	<0.0001
Model 2	1.00 (reference)	1.06 (0.95, 1.19)	1.36 (1.14, 1.62)	1.11 (1.05, 1.16)	<0.0001
Model 3	1.00 (reference)	1.08 (0.96, 1.21)	1.43 (1.20, 1.72)	1.13 (1.07, 1.18)	<0.0001
Model 4	1.00 (reference)	1.08 (0.96, 1.21)	1.32 (1.10, 1.58)	1.10 (1.05, 1.16)	0.0003
**Neuropathy**					
Cases/person-years	168/72602	366/117089	139/20451		

Incident rate per 1000 person-years	2.31	3.13	6.8		
Model 1	1.00 (reference)	1.34 (1.11, 1.61)	2.79 (2.21, 3.53)	1.36 (1.27, 1.45)	<0.0001
Model 2	1.00 (reference)	1.26 (1.04, 1.51)	2.41 (1.90, 3.06)	1.30 (1.21, 1.39)	<0.0001
Model 3	1.00 (reference)	1.19 (0.99, 1.43)	2.00 (1.57, 2.56)	1.23 (1.14, 1.32)	<0.0001
Model 4	1.00 (reference)	1.19 (0.99, 1.44)	1.87 (1.46, 2.39)	1.20 (1.12, 1.29)	<0.0001

[a] Model 1 adjusted for age (continuous), sex (male or female), ethnicity (White, Mixed, Asian, Black, Chinese, others, or unknown), educational attainment (college or university, vocational, upper secondary, lower secondary, others, or unknown), Townsend deprivation index (in quintiles), annual household income (<18000, 18000–30999, 31000–51999, 52000–100000, or >100000£ per year), and assessment centers (22 categories).

Model 2 adjusted for Model 1 plus smoking status (ever, former, or current smokers), alcohol intake (0, 0.1–4.9, 5.0–14.9, 15.0–19.9, 20.0–29.9, or ≥30.0 g/day), healthy diet score (in quintiles), BMI (<18.5, 18.5–24.9, 25.0–29.9, 30.0–34.9, or ≥35.0 kg/m^2).

Model 3 adjusted for Model 2 plus No. of long-term conditions (0, 1, 2, 3, 4, and ≥5).

Model 4 adjusted for Model 3 plus Diabetes duration (<1, 1–4, 5–9, ≥10 years), HbA1c (<53, ≥53 mmol/mol, missing), diabetes medication use (none, only oral medication, only insulin, or insulin and oral medication), lipid-lowering treatment, antihypertensive medication use, and aspirin use.

Table S9. Prevalence of Frailty Phenotype Components in Percentages.

	Overall	Frailty phenotype		
		Non-frail	Pre-frail	Frail
N	18062	6101(33.8%)	10073(55.8%)	1888(10.4%)
Weight loss	4843 (26.8%)	0 (0.0%)	3955 (39.3%)	888 (47.0%)
Exhaustion	3226 (17.9%)	0 (0.0%)	2031 (20.2%)	1195 (63.3%)
Low physical activity	3607 (20.0%)	0 (0.0%)	2347 (23.3%)	1260 (66.7%)
Slow gait speed	3487 (19.3%)	0 (0.0%)	1952 (19.4%)	1535 (81.3%)
Low grip strength	4500 (24.9%)	0 (0.0%)	3165 (31.4%)	1335 (70.7%)

Table S10. Association between Frailty Components and risk of diabetic microvascular complications [a].

	Model 1	Model 2	Model 3	Model 4	Model 5
**Overall microvascular complications**					
Weight loss	0.95 (0.88, 1.03)	0.96 (0.89, 1.03)	0.96 (0.89, 1.03)	0.99 (0.92, 1.07)	1.00 (0.93, 1.08)
Exhaustion	1.40 (1.29, 1.52)	1.32 (1.21, 1.43)	1.21 (1.11, 1.31)	1.17 (1.07, 1.27)	1.11 (1.02, 1.21)
Low physical activity	1.37 (1.27, 1.47)	1.26 (1.16, 1.36)	1.21 (1.12, 1.31)	1.18 (1.10, 1.28)	1.09 (1.01, 1.18)
Slow gait speed	1.81 (1.68, 1.95)	1.60 (1.48, 1.73)	1.47 (1.36, 1.59)	1.42 (1.31, 1.54)	1.37 (1.26, 1.48)
Low grip strength	1.27 (1.18, 1.37)	1.22 (1.13, 1.31)	1.18 (1.09, 1.27)	1.12 (1.04, 1.21)	1.08 (1.00, 1.16)
**Nephropathy**					
Weight loss	0.95 (0.86, 1.05)	0.96 (0.87, 1.06)	0.96 (0.87, 1.06)	0.97 (0.88, 1.07)	0.98 (0.89, 1.08)
Exhaustion	1.58 (1.43, 1.76)	1.45 (1.31, 1.61)	1.27 (1.14, 1.41)	1.25 (1.12, 1.39)	1.18 (1.06, 1.31)
Low physical activity	1.49 (1.36, 1.64)	1.31 (1.19, 1.45)	1.24 (1.13, 1.37)	1.22 (1.11, 1.35)	1.11 (1.00, 1.23)
Slow gait speed	2.13 (1.94, 2.33)	1.78 (1.62, 1.96)	1.56 (1.41, 1.72)	1.51 (1.37, 1.67)	1.44 (1.30, 1.60)
Low grip strength	1.28 (1.16, 1.40)	1.21 (1.10, 1.33)	1.15 (1.04, 1.26)	1.11 (1.01, 1.22)	1.05 (0.96, 1.16)
**Retinopathy**					
Weight loss	0.92 (0.82, 1.04)	0.91 (0.81, 1.02)	0.91 (0.81, 1.03)	1.00 (0.89, 1.12)	1.01 (0.89, 1.13)
Exhaustion	1.16 (1.02, 1.32)	1.14 (0.99, 1.30)	1.17 (1.02, 1.34)	1.09 (0.95, 1.25)	1.06 (0.92, 1.21)
Low physical activity	1.21 (1.07, 1.37)	1.18 (1.04, 1.34)	1.19 (1.05, 1.35)	1.14 (1.01, 1.29)	1.09 (0.96, 1.24)
Slow gait speed	1.28 (1.13, 1.45)	1.23 (1.08, 1.40)	1.27 (1.12, 1.45)	1.19 (1.05, 1.36)	1.14 (0.99, 1.31)
Low grip strength	1.31 (1.17, 1.47)	1.27 (1.13, 1.43)	1.29 (1.15, 1.45)	1.20 (1.07, 1.35)	1.18 (1.05, 1.33)
**Neuropathy**					
Weight loss	1.09 (0.92, 1.29)	1.09 (0.92, 1.29)	1.09 (0.92, 1.29)	1.15 (0.97, 1.37)	1.17 (0.98, 1.39)
Exhaustion	1.66 (1.39, 1.98)	1.56 (1.30, 1.86)	1.36 (1.13, 1.63)	1.30 (1.08, 1.56)	1.20 (1.00, 1.44)
Low physical activity	1.32 (1.11, 1.58)	1.21 (1.01, 1.44)	1.13 (0.94, 1.35)	1.09 (0.91, 1.30)	0.94 (0.78, 1.13)

Slow gait speed	2.44 (2.07, 2.87)	2.13 (1.79, 2.52)	1.86 (1.56, 2.22)	1.79 (1.50, 2.13)	1.77 (1.47, 2.12)
Low grip strength	1.27 (1.07, 1.50)	1.22 (1.03, 1.45)	1.16 (0.97, 1.38)	1.09 (0.91, 1.29)	1.02 (0.86, 1.21)

[a] Model 1 adjusted for age (continuous), sex (male or female), ethnicity (White, Mixed, Asian, Black, Chinese, others, or unknown), educational attainment (college or university, vocational, upper secondary, lower secondary, others, or unknown), Townsend deprivation index (in quintiles), annual household income (<18000, 18000–30999, 31000–51999, 52000–100000, or >100000£ per year), and assessment centers (22 categories).

Model 2 adjusted for Model 1 plus smoking status (ever, former, or current smokers), alcohol intake (0, 0.1–4.9, 5.0–14.9, 15.0–19.9, 20.0–29.9, or ≥30.0 g/day), healthy diet score (in quintiles), BMI (<18.5, 18.5–24.9, 25.0–29.9, 30.0–34.9, or ≥35.0 kg/m^2).

Model 3 adjusted for Model 2 plus No. of long-term conditions (0, 1, 2, 3, 4, and ≥5).

Model 4 adjusted for Model 3 plus Diabetes duration (<1, 1–4, 5–9, ≥10 years), HbA1c (<53, ≥53 mmol/mol, missing), diabetes medication use (none, only oral medication, only insulin, or insulin and oral medication), lipid-lowering treatment, antihypertensive medication use, and aspirin use at baseline.

Model 5 further mutually adjusted for other frailty components.

Table S11. Association between frailty and risk of overall diabetic microvascular complications by subgroups [a].

	N	Frailty phenotype			*P* for interaction
		Non-frail	Pre-frail	Frail	
**Baseline Age, years**					0.6454
< 60	7575	1.00 (reference)	1.11 (0.96, 1.28)	1.58 (1.29, 1.94)	
**≥ 60**	10487	1.00 (reference)	1.09 (1.00, 1.19)	1.51 (1.32, 1.72)	
**Sex**					0.8697
Female	6751	1.00 (reference)	1.10 (0.95, 1.26)	1.63 (1.36, 1.95)	
Male	11311	1.00 (reference)	1.10 (1.00, 1.20)	1.45 (1.26, 1.67)	
**Education**					
College or university	4855	1.00 (reference)	1.14 (0.97, 1.32)	1.68 (1.31, 2.16)	0.5070
Below college	12992	1.00 (reference)	1.08 (0.99, 1.18)	1.47 (1.3, 1.66)	
**Townsend deprivation index**					0.0827
Below median value (low deprivation)	9018	1.00 (reference)	1.15 (1.03, 1.28)	1.75 (1.49, 2.07)	
Above median value (high deprivation)	9018	1.00 (reference)	1.04 (0.93, 1.16)	1.37 (1.18, 1.58)	
**Annual household income, £**					0.0844
<31,000	9483	1.00 (reference)	1.01 (0.91, 1.12)	1.42 (1.24, 1.62)	
≥31,000	6158	1.00 (reference)	1.21 (1.05, 1.40)	1.64 (1.26, 2.13)	
**Smoking status**					
Never	7949	1.00 (reference)	1.11 (0.98, 1.25)	1.48 (1.24, 1.77)	0.5720
Former	8029	1.00 (reference)	1.07 (0.96, 1.19)	1.58 (1.35, 1.85)	
Current	2008	1.00 (reference)	1.18 (0.92, 1.51)	1.48 (1.07, 2.05)	
**Healthy diet score**					0.1687
Low	5445	1.00 (reference)	1.08 (0.93, 1.24)	1.37 (1.12, 1.68)	
Middle	4437	1.00 (reference)	1.10 (0.95, 1.28)	1.54 (1.24, 1.91)	
Hight	7734	1.00 (reference)	1.13 (1.00, 1.27)	1.64 (1.38, 1.95)	
**BMI, kg/m2**					0.9048
<30	8322	1.00 (reference)	1.13 (1.01, 1.26)	1.47 (1.20, 1.80)	
≥30	9740	1.00 (reference)	1.09 (0.98, 1.21)	1.59 (1.39, 1.82)	
**Diabetes duration, years**					0.8031

<1	4757	1.00 (reference)	1.03 (0.85, 1.24)	1.66 (1.26, 2.18)	
1–4.9	6152	1.01 (reference)	1.07 (0.93, 1.22)	1.38 (1.13, 1.69)	
5–9.9	4408	1.00 (reference)	1.16 (1.00, 1.34)	1.66 (1.34, 2.04)	
≥10	2745	1.01 (reference)	1.14 (0.97, 1.33)	1.46 (1.16, 1.83)	
**Diabetes medication use**					0.3402
None	8192	1.01 (reference)	1.03 (0.9, 1.17)	1.31 (1.06, 1.6)	
Only oral medication	8093	1.00 (reference)	1.07 (0.96, 1.2)	1.65 (1.42, 1.92)	
Insulin	1777	1.00 (reference)	1.35 (1.10, 1.65)	1.48 (1.12, 1.95)	
**HbA1c, mmol/mol**					0.9472
< 53.0	11019	1.00 (reference)	1.06 (0.96, 1.18)	1.49 (1.27, 1.73)	
≥ 53.0	5980	1.00 (reference)	1.14 (1.01, 1.29)	1.46 (1.23, 1.74)	
**No. of long-term conditions**					0.5334
0	2853	1.00 (reference)	1.18 (0.95, 1.47)	1.14 (0.67, 1.95)	
1	6084	1.00 (reference)	1.09 (0.95, 1.25)	1.44 (1.13, 1.83)	
2	4874	1.01 (reference)	1.01 (0.88, 1.17)	1.49 (1.21, 1.83)	
≥3	4251	1.00 (reference)	1.18 (1.01, 1.38)	1.66 (1.39, 1.99)	

[a] Cox proportional hazards models adjusted for age (continuous), sex (male or female), ethnicity (White, Mixed, Asian, Black, Chinese, others, or unknown), educational attainment (college or university, vocational, upper secondary, lower secondary, others, or unknown), Townsend deprivation index (in quintiles), annual household income (<18000, 18000–30999, 31000–51999, 52000–100000, or >100000£ per year), assessment centers (22 categories), smoking status (ever, former, or current smokers), alcohol intake (0, 0.1–4.9, 5.0–14.9, 15.0–19.9, 20.0–29.9, or ≥30.0 g/day), healthy diet score (in quintiles), BMI (<18.5, 18.5–24.9, 25.0–29.9, 30.0–34.9, or ≥35.0 kg/m^2), No. of long-term conditions (0, 1, 2, 3, 4, and ≥5), diabetes duration (<1, 1–4, 5–9, ≥10 years), HbA1c (<53, ≥53 mmol/mol, missing), diabetes medication use (none, only oral medication, only insulin, or insulin and oral medication), lipid-lowering treatment, antihypertensive medication use, and aspirin use. Each of the else groups were adjusted for all covariates expect itself.

Table S12. Sensitivity analyses of association between frailty phenotype and risk of diabetic microvascular complications (exclude individuals diagnosed any microvascular complications within 2 years of follow-up) [a]

	Frailty phenotype			Per score increment	*P* trend
	Non-frail	Pre-frail	Frail		
N	6013	9888	1808		
**Overall microvascular complications**					
Cases/person-years	1034/69040	1945/110587	536/18698		
Incident rate per 1000 person-years	14.98	17.59	28.67		
Hazard ratio (95% CI)	1.00 (reference)	1.08 (1.00, 1.17)	1.45 (1.30, 1.63)	1.12 (1.08, 1.15)	<0.0001
**Nephropathy**					
Cases/person-years	573/70767	1185/113728	360/19426		
Incident rate per 1000 person-years	8.1	10.42	18.53		
Hazard ratio (95% CI)	1.00 (reference)	1.13 (1.02, 1.25)	1.53 (1.33, 1.77)	1.14 (1.09, 1.19)	<0.0001
**Retinopathy**					
Cases/person-years	462/70927	795/114672	182/20087		
Incident rate per 1000 person-years	6.51	6.93	9.06		
Hazard ratio (95% CI)	1.00 (reference)	1.05 (0.93, 1.18)	1.31 (1.09, 1.57)	1.09 (1.04, 1.15)	0.0010
**Neuropathy**					
Cases/person-years	162/72060	335/116452	122/20203		
Incident rate per 1000 person-years (95% CI)	2.25	2.88	6.04		
Hazard ratio (95% CI)	1.00 (reference)	1.12 (0.93, 1.36)	1.71 (1.32, 2.21)	1.17 (1.09, 1.26)	<0.0001

[a] Exclusion of 353 individuals who diagnosed overall microvascular complications or died within 2 years of follow-up. Hazard ratios were adjusted for age (continuous), sex (male or female), ethnicity (White, Mixed, Asian, Black, Chinese, others, or unknown), educational attainment (college or university, vocational, upper secondary, lower secondary, others, or unknown), Townsend deprivation index (in quintiles), annual household income (<18000, 18000–30999, 31000–51999, 52000–100000, or >100000£ per year), assessment centers (22 categories), smoking status (ever, former, or current smokers), alcohol intake (0, 0.1–4.9, 5.0–14.9, 15.0–19.9, 20.0–29.9, or ≥30.0 g/day), healthy diet score (in quintiles), BMI (<18.5, 18.5–24.9, 25.0–29.9, 30.0–34.9, or ≥35.0 kg/m^2), No. of long-term conditions (0, 1, 2, 3, 4, and ≥5), diabetes duration (<1, 1–4, 5–9, ≥10 years), HbA1c (<53, ≥53 mmol/mol, missing), diabetes medication use (none, only oral medication, only insulin, or insulin and oral medication), lipid-lowering treatment, antihypertensive medication use, and aspirin use.

Table S13. Sensitivity analyses of association between frailty phenotype and risk of overall diabetic microvascular complications by sequentially adjusting for lipid profile, MAP, eGFR [a]

	Frailty phenotype			Per score increment	*P* trend
	**Non-frail**	**Pre-frail**	**Frail**		
N	6101	10073	1888		
**Overall microvascular complications**					
Model 4 + lipid profiles	1.00 (reference)	1.09 (1.01, 1.18)	1.50 (1.35, 1.68)	1.13 (1.09, 1.17)	<0.0001
Model 4 + lipid profiles + MAP	1.00 (reference)	1.08 (1.00, 1.17)	1.49 (1.33, 1.66)	1.13 (1.09, 1.16)	<0.0001
Model 4 + lipid profiles + MAP + eGFR	1.00 (reference)	1.08 (1.00, 1.16)	1.41 (1.26, 1.58)	1.11 (1.07, 1.14)	<0.0001
**Nephropathy**					
Model 4 + lipid profiles	1.00 (reference)	1.14 (1.03, 1.26)	1.57 (1.37, 1.81)	1.15 (1.11, 1.20)	<0.0001
Model 4 + lipid profiles + MAP	1.00 (reference)	1.13 (1.02, 1.24)	1.54 (1.34, 1.77)	1.15 (1.10, 1.19)	<0.0001
Model 4 + lipid profiles + MAP + eGFR	1.00 (reference)	1.11 (1.01, 1.23)	1.39 (1.21, 1.60)	1.11 (1.06, 1.15)	<0.0001
**Retinopathy**					
Model 4 + lipid profiles	1.00 (reference)	1.07 (0.96, 1.20)	1.31 (1.09, 1.57)	1.10 (1.04, 1.15)	0.0004
Model 4 + lipid profiles + MAP	1.00 (reference)	1.08 (0.96, 1.21)	1.32 (1.10, 1.59)	1.10 (1.05, 1.16)	0.0002
Model 4 + lipid profiles + MAP + eGFR	1.00 (reference)	1.08 (0.96, 1.21)	1.31 (1.09, 1.58)	1.10 (1.04, 1.16)	0.0004
**Neuropathy**					
Model 4 + lipid profiles	1.00 (reference)	1.19 (0.98, 1.43)	1.85 (1.45, 2.37)	1.20 (1.12, 1.29)	<0.0001
Model 4 + lipid profiles + MAP	1.00 (reference)	1.18 (0.98, 1.42)	1.83 (1.43, 2.34)	1.19 (1.11, 1.28)	<0.0001
Model 4 + lipid profiles + MAP + eGFR	1.00 (reference)	1.17 (0.97, 1.41)	1.80 (1.40, 2.31)	1.19 (1.10, 1.27)	<0.0001

[a] Sensitivity analyses were conducted by sequentially adjusting for lipid profiles (total cholesterol [TC], low-density lipoprotein [LDL], cholesterol and high-density lipoprotein [HDL] cholesterol, triglycerides [TG]), Mean arterial pressure (MAP), and estimated glomerular filtration rate (eGFR) at baseline base on Model 4.

Model 4, adjusted for age (continuous), sex (male or female), ethnicity (White, Mixed, Asian, Black, Chinese, others, or unknown), educational attainment (college or university, vocational, upper secondary, lower secondary, others, or unknown), Townsend deprivation index (in quintiles), annual household income (<18000, 18000–30999, 31000–51999, 52000–100000, or >100000£ per year), assessment centers (22 categories), smoking status (ever, former, or current smokers), alcohol intake (0, 0.1–4.9, 5.0–14.9, 15.0–19.9, 20.0–29.9, or ≥30.0 g/day), healthy

diet score (in quintiles), BMI (<18.5, 18.5–24.9, 25.0–29.9, 30.0–34.9, or ≥35.0 kg/m^2), No. of long-term conditions (0, 1, 2, 3, 4, and ≥5), diabetes duration (<1, 1–4, 5–9, ≥10 years), HbA1c (<53, ≥53 mmol/mol, missing), diabetes medication use (none, only oral medication, only insulin, or insulin and oral medication), lipid-lowering treatment, antihypertensive medication use, and aspirin use.

Table S14. Association between frailty phenotype and risk of overall diabetic microvascular complications using Fine & Gray models for competing risk [a]

	Frailty phenotype			Per score increment	*P* trend
	Non-frail	Pre-frail	Frail		
N	6101	10073	1888		
**Overall microvascular complications**	1.00 (reference)	1.06 (0.99, 1.15)	1.41 (1.26, 1.57)	1.11 (1.07, 1.14)	<0.0001
**Nephropathy**	1.00 (reference)	1.11 (1.01, 1.23)	1.47 (1.27, 1.70)	1.13 (1.08, 1.18)	<0.0001
**Retinopathy**	1.00 (reference)	1.05 (0.93, 1.18)	1.22 (1.01, 1.47)	1.07 (1.02, 1.13)	0.0089
**Neuropathy**	1.00 (reference)	1.15 (0.95, 1.38)	1.71 (1.33, 2.19)	1.16 (1.08, 1.25)	<0.0001

[a] Cox proportional hazards models adjusted for age (continuous), sex (male or female), ethnicity (White, Mixed, Asian, Black, Chinese, others, or unknown), educational attainment (college or university, vocational, upper secondary, lower secondary, others, or unknown), Townsend deprivation index (in quintiles), annual household income (<18000, 18000–30999, 31000–51999, 52000–100000, or >100000£ per year), assessment centers (22 categories), smoking status (ever, former, or current smokers), alcohol intake (0, 0.1–4.9, 5.0–14.9, 15.0–19.9, 20.0–29.9, or ≥30.0 g/day), healthy diet score (in quintiles), BMI (<18.5, 18.5–24.9, 25.0–29.9, 30.0–34.9, or ≥35.0 kg/m^2), No. of long-term conditions (0, 1, 2, 3, 4, and ≥5), diabetes duration (<1, 1–4, 5–9, ≥10 years), HbA1c (<53, ≥53 mmol/mol, missing), diabetes medication use (none, only oral medication, only insulin, or insulin and oral medication), lipid-lowering treatment, antihypertensive medication use, and aspirin use at baseline.

Table S15. Sensitivity analyses of association between frailty phenotype and risk of diabetic microvascular complications were conducted by using the Charlson comorbidity index as an alternative measure of comorbidities [a].

	Frailty phenotype			Per score increment	*P* trend
	Non-frail	Pre-frail	Frail		
N	6101	10073	1888		
**Overall microvascular complications**	1.00 (reference)	1.10 (1.02, 1.18)	1.52 (1.37, 1.7)	1.13 (1.10, 1.17)	<0.0001
**Nephropathy**	1.00 (reference)	1.15 (1.04, 1.27)	1.58 (1.38, 1.82)	1.15 (1.11, 1.20)	<0.0001
**Retinopathy**	1.00 (reference)	1.06 (0.95, 1.19)	1.27 (1.06, 1.52)	1.09 (1.03, 1.14)	0.0011
**Neuropathy**	1.00 (reference)	1.22 (1.01, 1.47)	1.99 (1.56, 2.54)	1.22 (1.14, 1.31)	<0.0001

[a] Cox proportional hazards models adjusted for age (continuous), sex (male or female), ethnicity (White, Mixed, Asian, Black, Chinese, others, or unknown), educational attainment (college or university, vocational, upper secondary, lower secondary, others, or unknown), Townsend deprivation index (in quintiles), annual household income (<18000, 18000–30999, 31000–51999, 52000–100000, or >100000£ per year), assessment centers (22 categories), smoking status (ever, former, or current smokers), alcohol intake (0, 0.1–4.9, 5.0–14.9, 15.0–19.9, 20.0–29.9, or ≥30.0 g/day), healthy diet score (in quintiles), BMI (<18.5, 18.5–24.9, 25.0–29.9, 30.0–34.9, or ≥35.0 kg/m^2), Charlson comorbidity index (0, 1, 2, and ≥3), diabetes duration (<1, 1–4, 5–9, ≥10 years), HbA1c (<53, ≥53 mmol/mol, missing), diabetes medication use (none, only oral medication, only insulin, or insulin and oral medication), lipid-lowering treatment, antihypertensive medication use, and aspirin use at baseline.

Table S16. Mediating effects of multimorbidity on the association of Frailty Phenotype with risk of diabetic microvascular complications among type 2 diabetes [a].

	Total effect	Direct effect	Proportion mediated	P value
**No. of long-term conditions**				
Overall microvascular complications	1.15 (1.11, 1.19)	1.19 (1.15, 1.23)	19.8% (14.5%, 26.5%)	<0.0001
Nephropathy	1.18 (1.13, 1.23)	1.24 (1.19, 1.29)	23.3% (17.3%, 30.5%)	<0.0001
Retinopathy	—*	—	—	—
Neuropathy	1.22 (1.14, 1.31)	1.30 (1.21, 1.39)	24.2% (15.8%, 35.1 %)	<0.0001
**Charlson comorbidity index**				
Overall microvascular complications	1.16 (1.12, 1.20)	1.19 (1.15, 1.23)	13.1% (9.7%, 17.5%)	<0.0001
Nephropathy	1.20 (1.15, 1.25)	1.24 (1.19, 1.29)	15.7% (11.8%, 20.5%)	<0.0001
Retinopathy	—	—	—	—
Neuropathy	1.26 (1.17, 1.35)	1.30 (1.21, 1.39)	12.2% (7.6%, 19.0 %)	<0.0001

[a] Data are HR (95% CI) or %; Mediation analyses were used. The covariates adjusted for in the mediation analyses included age, sex, ethnicity, educational attainment, Townsend deprivation index, annual household income, assessment centers, smoking status, alcohol intake, healthy diet score, BMI, diabetes duration, HbA1c, diabetes medication use, lipid-lowering treatment, antihypertensive medication use, and aspirin use.

*— means that the mediator was not intermediate to VPA proportion and outcome.
